# Soluble guanylate cyclase stimulators for heart failure: a network meta-analysis and subgroup analyses of reduced and preserved ejection fraction

**DOI:** 10.1186/s43044-024-00437-x

**Published:** 2024-01-24

**Authors:** Mohamed T. Abuelazm, Abdelrahman Attia, Mohamed Abdelnabi, Uzair Jafar, Omar Almaadawy, Mohamed A. Elzeftawy, Abdelrahman Mahmoud, Khaled Albakri, Basel Abdelazeem

**Affiliations:** 1 Faculty of Medicine, Tanta University, Tanta, Egypt; 2 Faculty of Medicine, Cairo University, Cairo, Egypt; 3 Department of Clinical Pharmacy, University of Michigan, Ann Arbor, MI USA; 4 Department of Medicine, King Edward Medical University, Lahore, Pakistan; 5 Department of Internal Medicine, MedStar Health, Baltimore, MD USA; 6 Faculty of Medicine, Minia University, Minia, Egypt; 7 Faculty of Medicine, Hashemite University, Zarqa, Jordan; 8 Department of Cardiology, West Virginia University, Morgantown, WV USA

**Keywords:** Soluble guanylate cyclase stimulators, sGC stimulator, Heart failure, Riociguat, Vericiguat, Review, Meta-analysis

## Abstract

**Background:**

Soluble guanylate cyclase (sGC) stimulators have been investigated for heart failure (HF) in several randomized controlled trials (RCTs). However, its place in the management guidelines of either HFrEF or HfpEF is still inconclusive.

**Methods:**

We conducted a network meta-analysis synthesizing RCTs investigating sGC for HF management, which were retrieved by systematically searching five databases until January 24th, 2023. Dichotomous outcomes were pooled using risk ratio (RR) along with confidence interval (CI).

**Results:**

Eight RCTs with a total of 7307 patients were included. Vericiguat 10 mg significantly reduced the composite cardiovascular (CVS) mortality and HF hospitalization in HF (RR: 0.88, 95% CI [0.79; 0.98]) and in HFrEF (RR: 0.87, 95% CI [0.78; 0.97]); however, it was not effective in HFpEF (RR: 0.69, 95% CI [0.15; 3.05]). Also, vericiguat 10 mg showed no difference compared to placebo regarding the incidence of all-cause mortality (RR: 0.96, 95% CI [0.84; 1.10]), any adverse events (AEs) (RR: 0.94, 95% CI [0.83; 1.07]), any serious AEs (RR: 0.91, 95% CI [0.81; 1.01]), and any AEs leading to drug discontinuation (RR: 1.14, 95% CI [0.92; 1.40]).

**Conclusion:**

Vericiguat 10 mg was effective in reducing the composite CVS mortality and HF hospitalization, with an acceptable safety profile. This was only observed in HFrEF patients, but not in HFpEF patients. However, our data regarding other agents (riociguat and praliciguat) and HFpEF can be underpowered, warranting further RCTs to clarify vericiguat 10 mg place in HFrEF management guidelines and to investigate sGC stimulators for HFpEF in large-scale trials.

**Supplementary Information:**

The online version contains supplementary material available at 10.1186/s43044-024-00437-x.

## Background

Heart failure (HF) is characterized by the inability of the heart to pump sufficient blood to meet the body's demands, leading to symptoms such as shortness of breath, fatigue, and swelling in the legs. It is a growing public health problem, affecting millions of people worldwide, and is associated with high rates of hospitalization and death [1, 2]. Soluble guanylate cyclase (sGC) stimulators are a class of drugs that increase the activity of sGC, an enzyme involved in nitric oxide signaling. sGC stimulators have been studied for their potential therapeutic benefits in several cardiovascular and pulmonary diseases, including HF and pulmonary arterial hypertension [3]. sGC stimulators work by increasing the levels of cyclic guanosine monophosphate in the body, leading to vasodilation and improved blood flow. This mechanism of action differs from traditional HF drugs, such as angiotensin-converting enzyme inhibitors (ACEIs), istaroxime, and beta-blockers (BBs), which target different pathways in the body [4, 5].

The efficacy of sGC stimulators in HF has been demonstrated in several clinical trials [2, 6–12]. For example, it was shown that the sGC stimulator vericiguat improved exercise capacity and reduced the risk of hospitalization for HF in patients with reduced ejection fraction (HFrEF) [2]. Another study found that vericiguat improved quality of life and reduced the risk of death and hospitalization in patients with HFrEF [13]. On the other hand, it was recently revealed that the sGC stimulator riociguat did not significantly improve exercise capacity or reduce the risk of hospitalization in patients with HF with preserved ejection fraction (HFpEF) [9].

However, more research is needed to fully understand the potential benefits and risks of sGC stimulators in HF and to determine the best ways to use these drugs in combination with other treatments. Further studies are also needed to determine the long-term effects of sGC stimulators on heart function and overall health outcomes. In this study, we aimed to evaluate the comparative efficacy and safety of sGC stimulators in patients HF, either with reduced or preserved ejection fraction. Also, we aim to conduct a thorough quality assessment of the current evidence and present a comprehensive network meta-analysis to guide clinical practice to the most effective sGC stimulator agent and dosage in HF.

## Methods

### Protocol registration

Our meta-analysis adheres to the recommended guidelines provided in the Preferred Reporting Items for Systematic Reviews and Meta-Analysis (PRISMA) statement [14] and the Cochrane Handbook for Systematic Reviews of Interventions [15]. The plan for conducting this study has been officially registered in The International Prospective Register of Systematic Reviews (PROSPERO) (CRD42023398846).

### Data sources and search strategy

Our search strategy comprised a comprehensive search of the Cochrane Central Register of Controlled Trials (CENTRAL, via The Cochrane Library), MEDLINE (via PubMed), Embase, SCOPUS, and Web of Science from inception till 24th January 2023 for any RCTs comparing sGC stimulators in HF with placebo or another sGC stimulators. The MeSH terms and relevant keywords for ("heart failure" OR "cardiac failure" OR HFrEF OR HFpEF) AND ("guanylate cyclase stimulator" OR riociguat OR vericiguat OR praliciguat) were used. The detailed search strategy can be found in the (Additional file 1: Table S1).

### Eligibility criteria

RCTs comparing sGC stimulators in HF with placebo or other sGC stimulators were included. Our primary outcome was the composite of cardiovascular mortality/HF hospitalization. The secondary outcomes included all-cause mortality, any adverse event, any serious adverse event, any adverse event leading to drug discontinuation, syncope, hypotension, and acute kidney injury (AKI).

We excluded the following types of studies from our analysis: research involving animals, preliminary studies, case reports, case series, clinical trials with only one treatment group, laboratory studies conducted in vitro, book chapters, editorial pieces, press articles, and conference abstracts.

### Study selection

All the eligible references were imported into the Covidence online software, and the duplicates were removed. U.J., O.A., M.A.E., and A.M. independently assessed the titles and abstracts of these articles, removing those not fulfilling our inclusion criteria. The full texts of the remaining articles were also screened independently. The discrepancies were resolved by B.A.

### Data extraction

Data from included studies were extracted by four authors (U.J., O.A., M.A.E., and A.M.) independently into a pre-piloted Excel sheet. B.A. rechecked the completed sheet and resolved any conflicts to ensure data accuracy. The following data items were extracted: study characteristics, including the study design, year of publication, study location, total participants, interventions (co-interventions, types, dosages, and treatment duration), and follow-up duration; population baseline data, including age, gender, and comorbidities; and outcome data.

### Risk of bias assessment

Four separate authors (U.J., O.A., M.A.E., and A.M.) evaluated the risk for bias in the studies included in our analysis using The Cochrane Collaboration's tool for assessing risk of bias, known as RoB 2.0 [16]. RoB 2.0 considers five specific areas: (1) bias resulting from the randomization process, (2) bias arising from deviations in the intended intervention, (3) bias related to missing outcome data, (4) bias in the measurement of outcomes, and (5) bias in the selection of reported results. In case of any disagreements, a consensus was reached among the authors after discussion.

### Statistical analysis

To analyze and combine the data, we utilized network analysis in the R software, employing the meta and net meta-packages. For dichotomous outcomes, we employed the risk ratio (RR) along with a 95% confidence interval (CI). The heterogeneity among the studies included in the analysis was assessed using the Chi-square and I-square (I^2^) tests. Data was considered heterogeneous if the Chi-square P-value was less than 0.1 and the I^2^ value exceeded 50%. Homogeneous data were pooled using a fixed-effect model, while heterogeneous data were pooled using a random-effect model. Furthermore, we conducted a subgroup analysis based on the type of HF, distinguishing between HFrEF and HFpEF.

## Results

### Search results and study selection

**Fig. 1:**
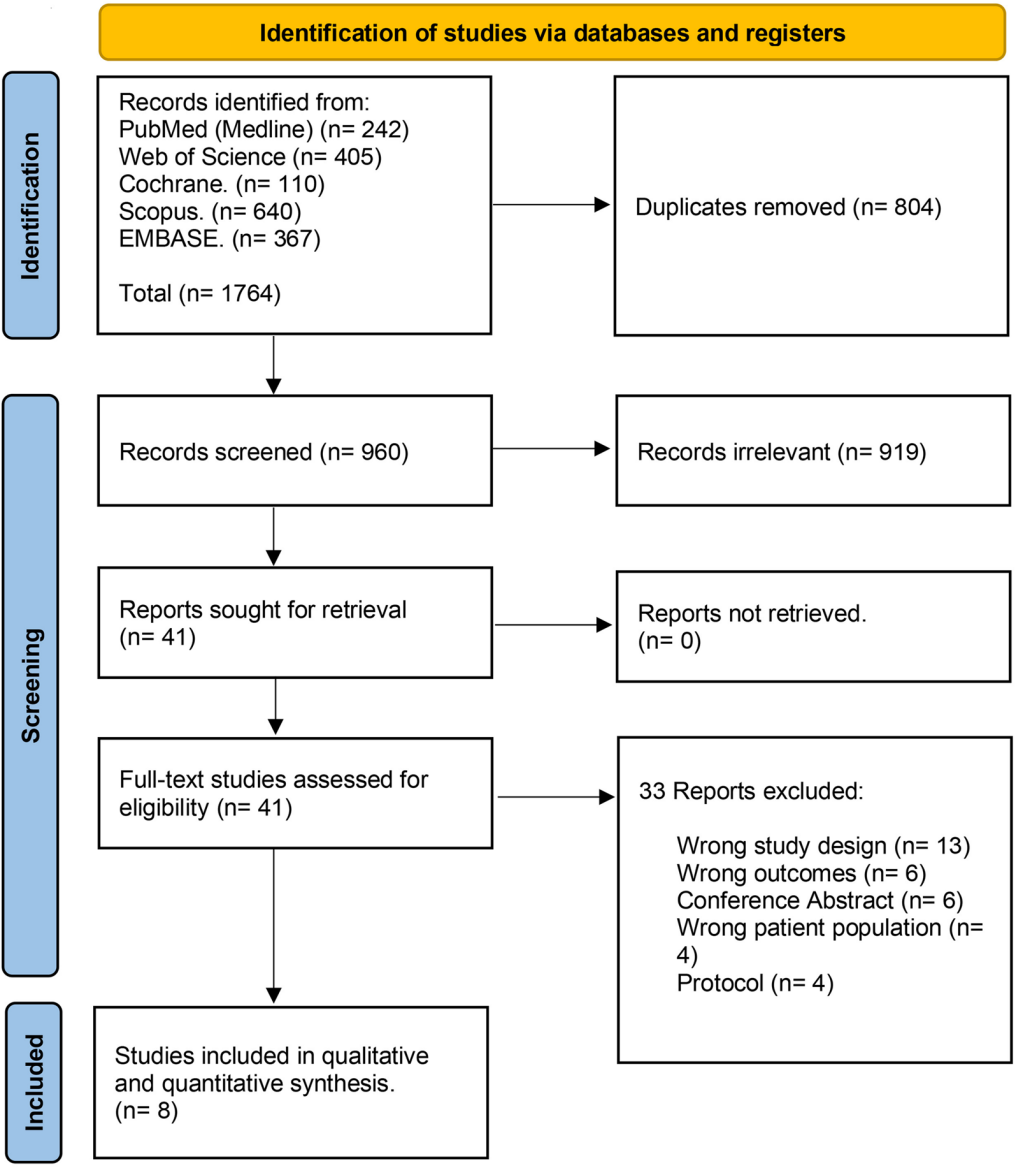
PRISMA flow chart of the screening process

After searching databases, a total of 1764 studies were retrieved for screening. Following the elimination of 804 duplicate studies and 919 studies that did not fulfill the inclusion criteria after the title and abstract screening, forty-one complete articles were thoroughly evaluated. Out of these, thirty-three records were determined to be ineligible and were subsequently excluded. This resulted in a final selection of eight RCTs that were eligible for both qualitative and quantitative analysis (Fig. 1).

### Characteristics of included studies

We included eight RCTs [2, 6–12] with a total of 7307 patients; 4086 in the sGC stimulator group and 3221 in the placebo group. Four trials used vericiguat as an intervention, three used riociguat, and only one used praliciguat. Five RCTs investigated HFpEF patients, and three investigated HFrEF patients. Detailed information about the summary and baseline characteristics of the included studies are found in (Tables 1 and 2).

**Table 1:** Summary characteristics of the included RCTs

Study ID	Study design	Country	Total participants	sGC stimulator				HF type/NYHA class	Primary outcome
				Drug	Dose	Times of administration	TTT duration		
Armstrong et al. 2020 (VITALITY-HFpEF) [8]	Phase IIb multicenter, double-blinded RCT	21 countries	789	Vericiguat	10 or 15 mg	Once daily	24 weeks	HFpEF/NYHA II & III	Kansas City Cardiomyopathy Questionnaire Physical Limitation Score (KCCQ PLS)
Armstrong et al. 2020 (VICTORIA) [2]	Phase III multicenter, double-blinded RCT	42 countries	5050	Vericiguat	10 mg	Once daily	N/A	HFrEF/NYHA II–IV	The composite of death from cardiovascular causes or first hospitalization for heart failure
Bonderman et al. 2013 (LEPHT) [10]	Phase IIb multicenter, double-blinded RCT	18 countries	201	Riociguat	0.5, 1, or 2 mg	Three times daily	16 weeks	HFrEF/NYHA II–IV	mPAP change
Bonderman et al. 2014 (DILATE-1) [7]	Phase II multicenter, double-blinded RCT	Austria, the Czech Republic, and Germany	39	Riociguat	0.5, 1, or 2 mg	Once daily	N/A	HFpEF	mPAP change
Dachs et al. 2022 (haemoDYNAMIC) [9]	Phase IIb multicenter, double-blinded RCT	Austria and Germany	114	Riociguat	0.5 mg (up-)titrated to 1.0 of 1.5 mg	Three times daily	30 weeks	HFpEF	CO change
Gheorghiade et al. 2015 (SOCRATES-Reduced) [9]	Multicenter, double-blinded RCT	Europe, North America, and Asia	456	Vericiguat	1.25, 2.5, 5, or 10 mg	Once daily	12 weeks	HFrEF/NYHA II–IV	NT-proBNP change
Pieske et al. 2017 (SOCRATES-PRESERVED) [12]	Multicenter, double-blinded RCT	United States and Germany	477	Vericiguat	1.25, 2.5, 5, or 10 mg	Once daily	12 weeks	HFpEF/NYHA II–IV	NT-proBNP change
Udelson et al. 2020 (CAPACITY HFpEF) [11]	Phase II multicenter, double-blinded RCT	United States and Canada	181	Praliciguat	40 mg	N/A	12 weeks	HFpEF/NYHA II–IV	Peak VO2 change

*N/A* not available, *HF* heart failure, *sGC* soluble guanylate cyclase, *TTT* treatment, *NYHA* New York Heart Association, *HFrEF* heart failure with reduced ejection fraction, *HFpEF* heart failure with preserved ejection fraction, *CO* cardiac output, *mPAP* mean pulmonary arterial pressure, *NT-proBNP* N-terminal pro-brain natriuretic peptide

**Table 2:** baseline characteristics of the participants

Study ID	Arm	Number of patients	Age (years), mean (SD)	Gender (male), N. (%)	BMI, mean (SD)	LVEF (%), mean (SD)	NT-proBNP, mean (SD)	eGFR, mean (SD)	Comorbidities, N. (%)							
									DM	AF	HTN	CKD	COPD	CAD		
Armstrong et al. 2020 (VITALITY-HFpEF) [8]	Vericiguat 10 mg	263	72.2 (9.7)	139 (52.9)	30.4 (6.0)	55.8 (8.3)	1700 (1732)	62.4 (20.6)	115 (43.7)	163 (62.0)	243 (92.4)	92 (35.0)	46 (17.5)	115 (43.7)		
	Vericiguat 15 mg	264	73.1 (9.1)	124 (47.0)	30.9 (6.2)	56.8 (7.9)	2826.5(1639)	59.1 (21.2)	120 (45.5)	164 (62.1)	243 (92.0)	110 (41.7)	57 (21.6)	120 (45.5)		
	Placebo	262	72.8 (9.4)	141 (53.8)	30.7 (6.0)	56.3 (7.9)	1867 (1763.2)	56.9 (20.0)	123 (46.9)	158 (60.3)	243 (92.7)	116 (44.3)	51 (19.5)	127 (48.5)		
Armstrong et al. 2020 (VICTORIA) [2]	Vericiguat 10 mg	2526	67.5 (12.2)	1921 (76)	27.7 (5.8)	29 (8.3)	3139.75 (1116.3)	61.3 (27)	1226 (48.6)	1098 (43.5)	2002 (79.3)	N/A	431 (17.1)	1511 (59.8)		
	Placebo	2524	67.2 (12.2)	1921 (76.1)	27.9 (6.1)	28.8 (8.3)	3099 (1068.1)	61.7 (27.3)	1143 (45.3)	1170 (46.4)	1993 (79)	N/A	436 (17.3)	1433 (56.8)		
Bonderman et al. 2013 (LEPHT) [10]	Riociguat 0.5 mg	26	57.2 (36–78)	26 (81)	29.2 (5.7)	27 (5)	1923 (1411.8)	72 (18.1)	13 (41)	3 (10)	N/A	N/A	N/A	N/A		
	Riociguat 1 mg	30	55.1 (28–74)	30 (91)	28.2 (4.6)	28.8 (4.6)	2417 (2730.4)	72.6 (23)	10 (30)	3 (9)	N/A	N/A	N/A	N/A		
	Riociguat 2 mg	55	59.3 (26–76)	55 (82)	28.9 (4.9)	28.4 (5.7)	2175 (2335.2)	65.1 (18.8)	30 (45)	9 (16)	N/A	N/A	N/A	N/A		
	Placebo	61	58.9 (25–79)	61 (88)	28.7 (5.8)	27.1 (5)	3000 (3472.2)	68.7 (19.9)	34 (49)	9 (15)	N/A	N/A	N/A	N/A		
Bonderman et al. 2014 (DILATE-1) [7]	Riociguat 0.5 mg	8	68.3 (48.0–80.0)	1 (13)	33.5 (22.9–44.9)	N/A	1765 (1323)	N/A	4 (50)	4 (50)	N/A	N/A	1 (13)	2 (25)		
	Riociguat 1 mg	7	65.3 (52.0–79.0)	3 (43)	31.0 (21.6–40.8)	N/A	852 (444)	N/A	3 (43)	3 (43)	N/A	N/A	0 (0)	1 (14)		
	Riociguat 2 mg	10	72.8 (59.0–83.0)	5 (50)	29.3 (23.5–33.4)	N/A	2537 (2394)	N/A	4 (40)	3 (30)	N/A	N/A	2 (20)	2 (20)		
	Placebo	11	75.1 (65.0–86.0)	6 (45)	30.2 (21.8–36.0)	N/A	2195 (1316)	N/A	5 (11)	6 (55)	N/A	N/A	4 (36)	1 (9)		
Dachs et al. 2022 (haemoDYNAMIC) [9]	Riociguat up-titrated 1.5 mg	58	70.6 (8.0)	12 (20.7)	32.1 (6.4)	61.0 (6.7)	819.6	63.4 (21.9)	16 (27.6)	36 (62.1)	38 (65.5)	27 (46.6)	5 (8.6)	7 (12.1)		
	Placebo	56	72.1 (8.5)	19 (33.9)	30.3 (6.4)	60.1 (6.0)	1051.3	61.7 (20.1)	16 (28.6)	37 (66.1)	34 (60.7)	27 (48.2)	5 (8.9)	8 (14.3)		
Gheorghiade et al. 2015 (SOCRATES-Reduced) [6]	Vericiguat 1.25 mg	91	68 (13)	70 (76.9)	28 (6)	29.5 (8.6)	3529 (3562)	57.2 (21.0)	36 (39.6)	32 (35.2)	71 (78.0)	35 (38.5)	N/A	46 (50.5)		
	Vericiguat 2.5 mg	91	68 (12)	72 (79.1)	28 (5)	29.2 (8.2)	2921 (2452)	56.9 (19.1)	54 (59.3)	30 (33.0)	70 (76.9)	41 (45.1)		57 (62.6)		
	Vericiguat 5 mg	91	67 (12)	74 (81.3)	29 (5)	31.5 (8.5)	4229 (5248)	60.1 (20.2)	39 (42.9)	30 (33.0)	68 (74.7)	37 (40.7)		42 (46.2)		
	Vericiguat 10 mg	91	69 (12)	77 (84.6)	28 (5)	29.3 (8.3)	4511 (5197)	60.0 (19.6)	49 (53.8)	32 (35.2)	78 (85.7)	35 (38.5)		46 (50.5)		
	Placebo	92	67 (13)	73 (79.3)	27 (5)	28.6 (8.5)	4239 (3577)	57.8 (17.4)	41 (44.6)	30 (32.6)	70 (76.1)	38 (41.3)		51 (55.4)		
Pieske et al. 2017 (SOCRATES-PRESERVED) [12]	Vericiguat 1.25 mg	96	74 (10)	45 (46.9)	29.6 (6.5)	56.3 (5.3)	1376.7 (1631)	52.8 (23.0)	48 (50.0)	41 (42.7)	86 (89.6)	48 (50.0)	N/A	N/A		
	Vericiguat 2.5 mg	96	72 (11)	53 (55.2)	30.7 (6.3)	57 (7.5)	1268 (1413.5)	57.4 (20.8)	46 (47.9)	40 (41.7)	85 (88.5)	34 (35.4)	N/A	N/A		
	Vericiguat 5 mg	96	74 (8)	53 (55.2)	30.1 (5.6)	57.7 (6.8)	1700 (2289)	54.2 (17.3)	47 (49.0)	38 (39.6)	86 (89.6)	33 (34.4)	N/A	N/A		
	Vericiguat 10 mg	96	73 (10)	52 (54.2)	30.4 (5.0)	56.3 (5.3)	1527 (1643)	57.4 (19.3)	44 (45.8)	36 (37.5)	90 (93.8)	38 (39.6)	N/A	N/A		
	Placebo	93	74 (9)	47 (50.5)	30.1 (6.5)	57.3 (6.8)	1360 (1540)	52.3 (20.6)	47 (50.5)	35 (37.6)	85 (91.4)	43 (46.2)	N/A	N/A		
Udelson et al. 2020 (CAPACITY HFpEF) [11]	Praliciguat 40 mg	91	70.7 (9.2)	56 (61.5)	34.1 (6.1)	61.9 (7.5)	1516 (3221)	65.4 (20.0)	46 (50.5)	14 (15.4)	90 (98.9)	24 (26.4)	N/A	36 (39.6)		
	Placebo	90	70.1 (9.0)	50 (55.6)	34.7 (7.3)	59.8 (9.3)	1792 (3864)	68.6 (21.7)	50 (55.6)	17 (18.9)	87 (96.7)	14 (15.6)		35 (38.9)		

Study ID	Arm	Number of patients	Age (years), mean (SD)	Gender (male), N. (%)	BMI, mean (SD)	LVEF (%), mean (SD)	NT-proBNP, mean (SD)	eGFR, mean (SD)	Concomitant medication, N. (%)							
									Diuretics	ACEIs	ARBs	BBs	CBBs	Anti-coagulants	Anti-platelets	MRAs
Armstrong et al. 2020 (VITALITY-HFpEF) [8]	Vericiguat 10 mg	263	72.2 (9.7)	139 (52.9)	30.4 (6.0)	55.8 (8.3)	1700 (1732)	62.4 (20.6)	N/A	N/A	N/A	N/A	N/A	N/A	N/A	N/A
	Vericiguat 15 mg	264	73.1 (9.1)	124 (47.0)	30.9 (6.2)	56.8 (7.9)	2826.5(1639)	59.1 (21.2)	N/A	N/A	N/A	N/A	N/A	N/A	N/A	c
	Placebo	262	72.8 (9.4)	141 (53.8)	30.7 (6.0)	56.3 (7.9)	1867 (1763.2)	56.9 (20.0)	N/A	N/A	N/A	N/A	N/A	N/A	N/A	N/A
Armstrong et al. 2020 (VICTORIA) [2]	Vericiguat 10 mg	2526	67.5 (12.2)	1921 (76)	27.7 (5.8)	29 (8.3)	3139.75 (1116.3)	61.3 (27)	N/A	1847 (73.3)	2349 (93.2)	N/A	N/A		N/A	
	Placebo	2524	67.2 (12.2)	1921 (76.1)	27.9 (6.1)	28.8 (8.3)	3099 (1068.1)	61.7 (27.3)	N/A	1853 (73.6)	2342 (93)	N/A	N/A		N/A	
Bonderman et al. 2013 (LEPHT) [10]	Riociguat 0.5 mg	26	57.2 (36–78)	26 (81)	29.2 (5.7)	27 (5)	1923 (1411.8)	72 (18.1)	29 (91)	21 (66)	10 (31)	32 (100)	N/A	15 (47)	N/A	23 (72)
	Riociguat 1 mg	30	55.1 (28–74)	30 (91)	28.2 (4.6)	28.8 (4.6)	2417 (2730.4)	72.6 (23)	31 (94)	25 (76)	8 (24)	32 (94)	N/A	16 (49)	N/A	26 (79)
	Riociguat 2 mg	55	59.3 (26–76)	55 (82)	28.9 (4.9)	28.4 (5.7)	2175 (2335.2)	65.1 (18.8)	62 (93)	50 (75)	20 (30)	61 (91)	N/A	38 (57)	N/A	51 (76)
	Placebo	61	58.9 (25–79)	61 (88)	28.7 (5.8)	27.1 (5)	3000 (3472.2)	68.7 (19.9)	66 (96)	46 (67)	19 (28)	62 (90)	N/A	33 (48)	N/A	53 (77)
Bonderman et al. 2014 (DILATE-1) [7]	Riociguat 0.5 mg	8	68.3 (48.0–80.0)	1 (13)	33.5 (22.9–44.9)	N/A	1765 (1323)	N/A	6 (76)	2 (25)	5 (63)	5 (63)	3 (38)	6 (75)	N/A	2 (25)
	Riociguat 1 mg	7	65.3 (52.0–79.0)	3 (43)	31.0 (21.6–40.8)	N/A	852 (444)	N/A	5 (72)	6 (86)	1 (14)	6 (86)	2 (29)	5 (71)	N/A	6 (86)
	Riociguat 2 mg	10	72.8 (59.0–83.0)	5 (50)	29.3 (23.5–33.4)	N/A	2537 (2394)	N/A	5 (50)	6 (60)	2 (20)	8 (80)	5 (50)	5 (50)	N/A	8 (80)
	Placebo	11	75.1 (65.0–86.0)	6 (45)	30.2 (21.8–36.0)	N/A	2195 (1316)	N/A	9 (82)	3 (27)	5 (45)	10 (91)	6 (55)	7 (64)	N/A	7 (64)
Dachs et al. 2022 (haemoDYNAMIC) [9]	Riociguat up-titrated 1.5 mg	58	70.6 (8.0)	12 (20.7)	32.1 (6.4)	61.0 (6.7)	819.6	63.4 (21.9)	47 (81)	42 (72.4)	43 (74.1)	N/A	34 (58.6)	40 (79)	8(13.8)	
	Placebo	56	72.1 (8.5)	19 (33.9)	30.3 (6.4)	60.1 (6.0)	1051.3	61.7 (20.1)	49 (87.5)	40 (71.4)	42 (75.0)	N/A	31 (55.4)	42 (75)	13 (23.2)	
Gheorghiade et al. 2015 (SOCRATES-Reduced) [6]	Vericiguat 1.25 mg	91	68 (13)	70 (76.9)	28 (6)	29.5 (8.6)	3529 (3562)	57.2 (21.0)	82 (90.1)	59 (64.8)	17 (18.7)	77 (84.6)	N/A	N/A	N/A	51 (56)
	Vericiguat 2.5 mg	91	68 (12)	72 (79.1)	28 (5)	29.2 (8.2)	2921 (2452)	56.9 (19.1)	87 (95.6)	60 (65.9)	18 (19.8)	79 (86.8)			N/A	61 (67)
	Vericiguat 5 mg	91	67 (12)	74 (81.3)	29 (5)	31.5 (8.5)	4229 (5248)	60.1 (20.2)	84 (92.3)	53 (58.2)	29 (31.9)	86 (94.5)			N/A	58 (63.7)
	Vericiguat 10 mg	91	69 (12)	77 (84.6)	28 (5)	29.3 (8.3)	4511 (5197)	60.0 (19.6)	91 (100.0)	56 (61.5)	19 (20.9)	86 (94.5)			N/A	64 (70.3)
	Placebo	92	67 (13)	73 (79.3)	27 (5)	28.6 (8.5)	4239 (3577)	57.8 (17.4)	86 (93.5)	52 (56.5)	21 (22.8)	83 (90.2)			N/A	50 (54.3)
Pieske et al. 2017 (SOCRATES-PRESERVED) [12]	Vericiguat 1.25 mg	96	74 (10)	45 (46.9)	29.6 (6.5)	56.3 (5.3)	1376.7 (1631)	52.8 (23.0)	91 (94.8)	42 (43.8)	32 (33.3)	73 (76.0)	38 (39.6)	N/A	N/A	37 (38.5)
	Vericiguat 2.5 mg	96	72 (11)	53 (55.2)	30.7 (6.3)	57 (7.5)	1268 (1413.5)	57.4 (20.8)	85 (88.5)	41 (42.7)	33 (34.4)	76 (79.2)	40 (41.7)		N/A	34 (35.4)
	Vericiguat 5 mg	96	74 (8)	53 (55.2)	30.1 (5.6)	57.7 (6.8)	1700 (2289)	54.2 (17.3)	88 (92.6)	33 (34.7)	31 (32.6)	73 (76.8)	30 (31.6)		N/A	35 (36.8)
	Vericiguat 10 mg	96	73 (10)	52 (54.2)	30.4 (5.0)	56.3 (5.3)	1527 (1643)	57.4 (19.3)	90 (93.8)	35 (36.5)	34 (35.4)	82 (85.4)	33 (34.4)		N/A	33 (34.4)
	Placebo	93	74 (9)	47 (50.5)	30.1 (6.5)	57.3 (6.8)	1360 (1540)	52.3 (20.6)	85 (91.4)	40 (43.0)	32 (34.4)	76 (81.7)	30 (32.3)		N/A	39 (41.9)
Udelson et al. 2020 (CAPACITY HFpEF) [11]	Praliciguat 40 mg	91	70.7 (9.2)	56 (61.5)	34.1 (6.1)	61.9 (7.5)	1516 (3221)	65.4 (20.0)	17 (18.7)	27 (29.7)	2 (2.2)	39 (42.9)	28 (30.8)	21 (23.1)	63 (69.2)	N/A
	Placebo	90	70.1 (9.0)	50 (55.6)	34.7 (7.3)	59.8 (9.3)	1792 (3864)	68.6 (21.7)	17 (18.9)	36 (40.0)	3 (3.3)	24 (26.7)	26 (28.9)	17 (18.9)	60 (66.7)	N/A

*N/A* not available, *SD* standard deviation, *N* number, *BMI* basal metabolic index, *LVEF* left ventricular ejection fraction, *NT-proBNP* N-terminal pro-brain natriuretic peptide, *eGFR* estimated glomerular filtration rate, *DM* diabetes mellitus, *AF* atrial fibrillation, *HTN* hypertension, *CKD* chronic kidney disease, *COPD* chronic obstructive pulmonary disease, *CAD* coronary artery disease, *ACEIs* angiotensin converting enzyme inhibitors, *ARBs* angiotensinogen receptor blockers, *BBs* beta blockers, *CCBs* calcium channel blockers, *MRAs* mineralocorticoid antagonists

### Risk of bias

**Fig. 2:**
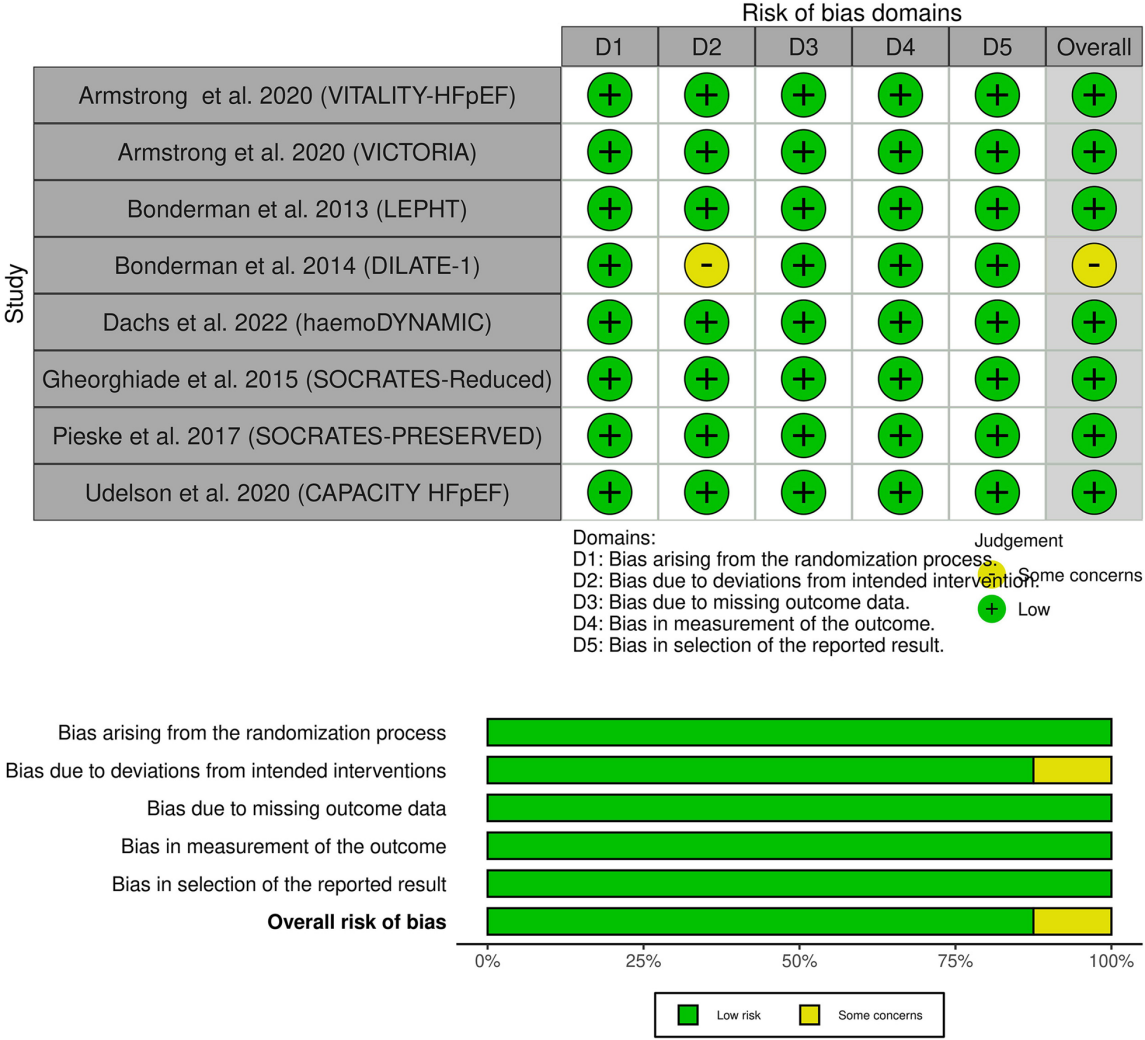
Quality assessment of the risk of bias in the included trials. The upper panel presents a schematic representation of risks (low = red, unclear = yellow, and high = red) for specific types of biases of each of the studies in the review. The lower panel presents risks (low = red, unclear = yellow, and high = red) for the subtypes of biases of the combination of studies included in this review

All included RCTs showed an overall low risk of bias, except for Dilate-1 [7], which showed some concerns due to concerns about deviation from the intended intervention. More details can be obtained from (Fig. 2).

### Efficacy outcome (the composite of cardiovascular mortality/HF hospitalization)

**Fig. 3:**
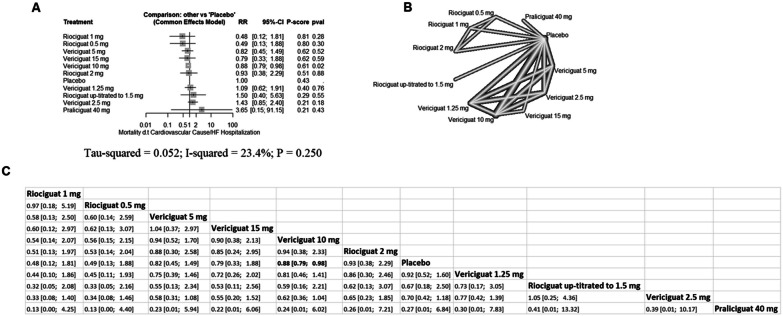
Network meta-analysis of the composite of cardiovascular mortality/HF hospitalization for the general HF population (**A** forest plot, **B** network plot, **C** rank table), *RR* risk ratio, *CI* confidence interval

Vericiguat 10 mg significantly decreased the risk of cardiovascular mortality/HF hospitalization (RR = 0.88 with 95% CI [0.79; 0.98], P = 0.02). However, the remining comparisons showed no significant difference (Fig. 3). Pooled studies were homogenous (I^2^ = 23.4%, p = 0.25).

**Fig. 4:**
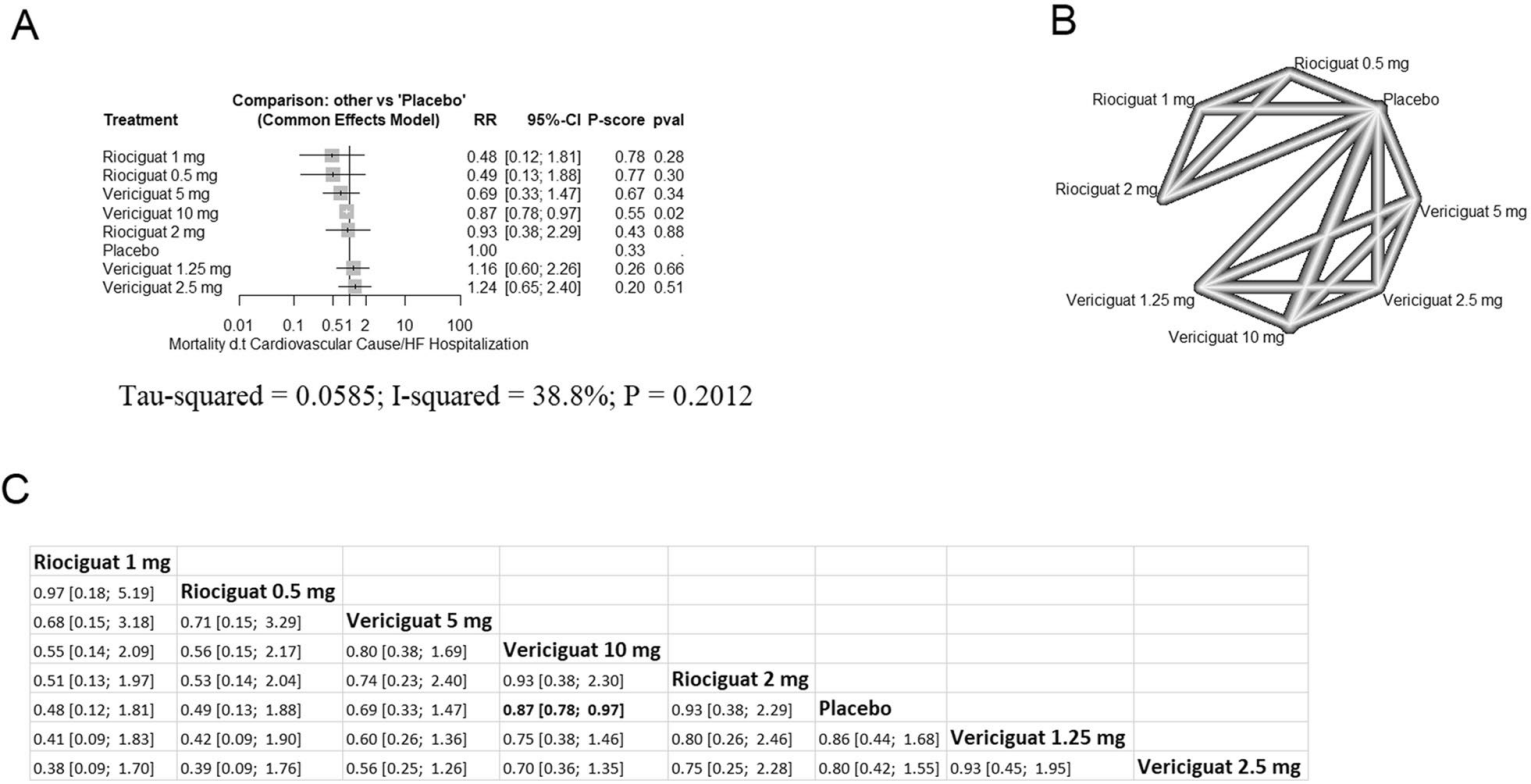
Network meta-analysis of the composite of cardiovascular mortality/HF hospitalization for HFrEF (**A** forest plot, **B** network plot, **C** rank table), *RR* risk ratio, *CI* confidence interval

In HFrEF patients, vericiguat 10 mg significantly decreased the risk of cardiovascular mortality/HF hospitalization (RR = 0.87 with 95% CI [0.78; 0.97], P = 0.02). However, the remining comparisons showed no significant difference (Fig. 4). Pooled studies were homogenous (I^2^ = 38.8%, p = 0.21). In HFpEF patients, all comparisons showed non-significant differences (Additional file 1: Fig. S1).

### Safety outcomes

Riociguat (0.5 mg, 1 mg, and up-titrated to 1.5 mg), praliciguat 40 mg, and vericiguat (1.25 mg, 2.5 mg, 5 mg, 10 mg, and 15 mg) showed no difference compared to placebo regarding all-cause mortality (Additional file 1: Fig. S2), any adverse event (Additional file 1: Fig. S3), any serious adverse event (Additional file 1: Fig. S4), any adverse event leading to drug discontinuation (Additional file 1: Fig. S5), syncope (Additional file 1: Fig. S6), and AKI (Additional file 1: Fig. S7). However, praliciguat 40 mg showed a higher risk of hypotension than placebo (RR: 18.43 with 95% CI [1.05; 324.20], P = 0.05) (Additional file 1: Fig. S8). Also, praliciguat 40 mg has higher risk of hypotension than vericiguat 5 mg, vericiguat 2.5 mg, vericiguat 1.25 mg, vericiguat 15 mg as shown in rank table (Additional file 1: Fig. S8).

Pooled studies were homogenous in all-cause mortality (I^2^ = 23%, p = 0.23), any adverse event (I^2^ = 0%, p = 0.95), any serious adverse event (I^2^ = 0%, p = 0.94), any adverse event leading to drug discontinuation (I^2^ =  = 0%, p = 0.95), hypotension (I^2^ = 20%, p = 0.27), syncope (I^2^ = 0%, p = 0.96), and AKI (I^2^ = 0%, p = 0.7).

**Fig. 5:**
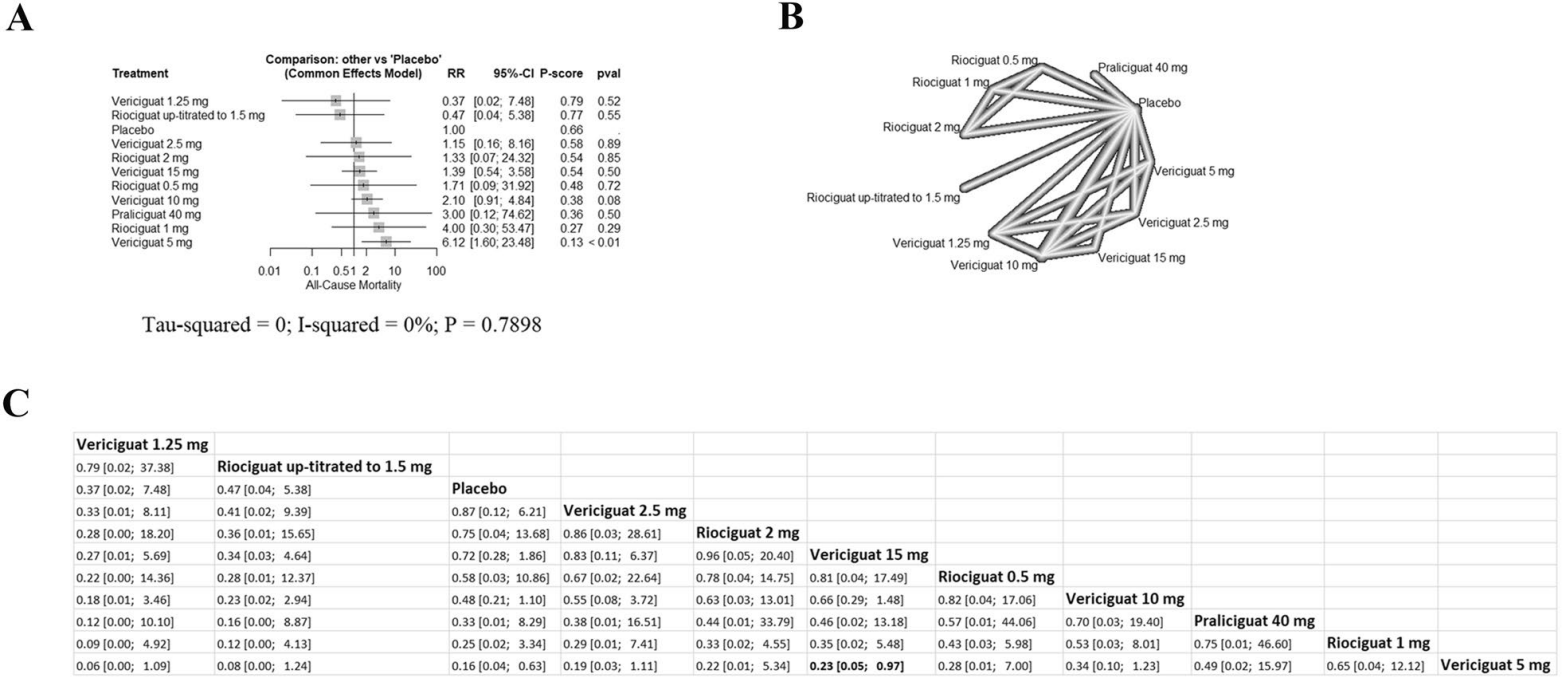
Network meta-analysis of the composite of all-cause mortality for the general HF population (**A** forest plot, **B** network plot, **C** rank table), *RR* risk ratio, *CI* confidence interval

Subgroup analysis showed similar findings with no difference between s GC stimulators and placebo in any adverse event (Additional file 1: Figs. S9, S10), any serious adverse event (Additional file 1: Figs. S11, S12), any adverse event leading to drug discontinuation (Additional file 1: Figs. S13, S14), syncope (Additional file 1: Figs. S15, S16), and AKI (Additional file 1: Figs. S17, S18). However, among patients with HFpEF, vericiguat 5 mg increased the risk of all-cause mortality in comparison with placebo (RR: 6.12 with 95% CI [1.60; 23.48], P < 0.01) (Fig. 5), with no difference in patients with HFrEF (Additional file 1: Fig. S19). Also, praliciguat 40 mg increased the risk of hypotension in comparison with placebo in patients with HFpEF (RR: 18.43 with 95% CI [1.05; 324.20]) (Additional file 1: Fig. S20), with no difference in patients with HFrEF (Additional file 1: Fig. S21).

## Discussion

sGC stimulators have emerged as a potential treatment for HF due to their ability to stimulate the production of cyclic guanosine monophosphate, which is an impaired pathway in those patients [17, 18]. Despite being tested for safety and efficacy in several clinical trials, only a few studies have reviewed and analyzed the reported data. In this systematic review and meta-analysis, we synthesized evidence from eight RCTs conducted between 2013 and 2022 with a total of 7307 HF patients. Our analysis shows that only vericiguat at a dose of 10 mg significantly reduced the risk of composite cardiovascular mortality and HF hospitalization in patients with HF, which was only sustained in the HFrEF subgroup, with no effect in HFpEF patients. Vericiguat also showed to be relatively safe, with only an increased risk of all-cause mortality in HFpEF patients at 5 mg. However, riociguat and praliciguat did not show different effects from placebo on the composite cardiovascular mortality and HF hospitalization, but an increased risk of hypotension in general HF and HFpEF patients was observed in the praliciguat group at the dose of 40 mg.

Vericiguat showed to have the best outcomes despite the variations in the follow-up duration and HF type between the included trials. Among the tested range of doses (1.25–15 mg), 10 mg was only effective when administered once per day. Furthermore, our subgroup analysis showed that vericiguat 10 mg had positive outcomes mainly in HFrEF, a type of HF that represents approximately 50% of all HF cases and has a high mortality rate of approximately 75% [19, 20]. Conversely, HFpEF patients did not seem to benefit from vericiguat. Our findings may have been influenced by the large subgroup population of HFrEF patients included in the VICTORIA trial [2]. Therefore, further research with a larger population of HFpEF patients is recommended to confirm our findings. However, a recent network meta-analysis showed that other pharmacotherapies such as sodium-glucose transporter sodium-glucose cotransporter-2 (SGLT2) inhibitors, angiotensin receptor-neprilysin inhibitors (ARNIs), and mineralocorticoid receptor antagonists (MRAs) significantly reduced HF hospitalization in HFpEF, which could help this particular patient population unlike sGC stimulators [21].

In HF, N-terminal pro-B-type natriuretic peptide (NT-proBNB) is released from the cardiac myocytes in response to the increased stretching/stress of the cardiac wall, and therefore it is considered a gold standard biomarker of HF [22–24]. NT-proBNB levels in the blood are also used to monitor the effectiveness of certain HF medications such as beta-blockers, ACE inhibitors, and diuretics [23, 25]. Pieske et al. in the SOCRATES-PRESERVED trial and Gheorghiade et al. in the SOCRATES-REDUCED trial [6, 12] investigated the effect of vericiguat on the baseline change of log-transformed NT-proBNB and found no statistically significant reduction (improvement) in the log-transformed NT-proBNB levels at 12 weeks post-treatment. Dachs et al. [9] also reported no improvement in NT-proBNB levels in HFpEF patients 26 weeks after treatment with riociguat. Left atrial volume (LAV) was another outcome measured by Pieske et al. [12] through echocardiography as an indicator of left ventricular filling pressure (LVFP). Vericiguat did not show a significant effect on LAV compared with placebo.

Moreover, Udelson et al., Dachs et al., Bonderman et al., and Armstrong et al. [7–9, 11] utilized the 6-min-walk test (6MWT) to assess the HF patients’ exercise tolerance and monitor their response to sGC stimulators treatment. They reported no statistically significant change in 6MWT from baseline after treatment with praliciguat (40 mg), riociguat (0.5 mg (up-titrated to 1.0 or 1.5 mg)), riociguat (0.5, 1 and 2 mg), and vericiguat (10 or 15 mg), respectively. Additionally, Pieske et al. and Bonderman et al. [7, 12] evaluated the effects of vericiguat and riociguat, respectively, on the quality of life of HF patients using the 5-dimension EuroQol questionnaire (EQ-5D) and the scores did not show significant improvement compared with placebo.

Regarding the safety of sGC stimulators use in HF patients, when compared with a placebo, sGC stimulators were safe and well-tolerable with no reported serious adverse events, adverse events leading to drug discontinuation, or any adverse events, including syncope and AKI. However, hypotension was a common adverse event with praliciguat use in HFpEF patients. Also, while all sGC stimulators did not show an increase in the incidence of all-cause mortality, vericiguat 5 mg showed a higher risk than placebo in HFpEF patients. Similarly, BBs, MRAs, ACEIs, angiotensin receptor blockers (ARBs), ARNIs, and SGLT2 inhibitors were not effective on all-cause mortality in a recent network meta-analysis evaluating pharmacotherapies in HFpEF patients [21]. Conversely, in another recent meta-analysis, vericiguat showed to significantly reduce all-cause-mortality in HFrEF patients when combined with ARNIs, BBs, and MRAs, despite being not significantly different from SGLT2 inhibitors and omecamtiv-mecarbil [26].

Moreover, The PARADIGM-HF and DAPA-HF trials; on the other hand, have neprilysin inhibition (sacubitril/valsartan) and SGLT2 inhibition (Dapagliflozin), respectively [27, 28]. These trials have shown significant benefits in reducing mortality, hospitalizations, and improving symptoms in patients with HF, providing additional therapeutic options beyond traditional therapies. The differential outcomes between trials of sGC stimulators and those like PARADIGM-HF and DAPA-HF underscore the complex pathophysiology of HF and the need for a multifaceted approach to its management [27, 28].

Therefore, further research to investigate the effect of sGC stimulators on both HFrEF and HFpEF outcomes when combined with other HF medications is still warranted, which may allow for a more reliable conclusion regarding the position of sGC stimulators in HF management guidelines.

### Strengths and limitations

This a network meta-analysis of double-blinded, multinational/centric RCTs, which strengthens the quality of our evidence and increases the generalizability of our study, with no identified heterogeneity of the data. However, our analysis has a few limitations. First, the population size of HFpEF patients, was small, which made it challenging to draw strong conclusions. Second, only one trial in our analysis evaluated the safety and efficacy of praliciguat in HF patients limiting the power of our data regarding its true effect. Third, we could not include some efficacy outcomes, such as NT-proBNB, 6MWT, LAV, and EQ-5D questionnaire in our analysis due to the lack of or the significant variation in the reported data. Finally, the follow-up duration among the included trials was not long enough to determine the long-term safety and efficacy of sGC stimulators in HF patients.

## Conclusions

In this network meta-analysis investigating sGC stimulators for HF management, only vericiguat 10 mg was effective in reducing the incidence of the composite cardiovascular mortality and HF hospitalization, with an acceptable safety profile. Also, this was only observed in patients with HFrEF, but not in patients with HFpEF. However, this observation is mainly weighted by the VICTORIA trial, constituting 69.2% of our analyzed sample size, which investigated vericiguat 10 mg in patients with HFrEF. Therefore, our data regarding other agents (riociguat and praliciguat) and HFpEF analysis can be underpowered. This warrants further RCTs to clarify vericiguat 10 mg place in HFrEF management guidelines by conducting head-to-head comparisons or combinations with other approved HF drugs and to investigate sGC stimulators for HFpEF in large-scale adequately designed trials.

### Supplementary Information

**Additional file 1.** Supplementary tables and figures.

## Data Availability

Not applicable.

## Abbreviations

HF: Heart failure
sGC: Soluble guanylate cyclase
ACEIs: Angiotensin-converting enzyme inhibitors
BBs: Beta-blockers
HFrEF: Heart failure with reduced ejection fraction
HFpEF: Heart failure with preserved ejection fraction
RCTs: Randomized controlled trials
AKI: Acute kidney injury
RR: Risk ratio
CI: Confidence interval
I^2^: I-square
SGLT2: Sodium-glucose transporter sodium-glucose cotransporter-2
ARNIs: Angiotensin receptor-neprilysin inhibitors
MRAs: Mineralocorticoid receptor antagonists
NT-proBNB: N-terminal pro-B-type natriuretic peptide
LAV: Left atrial volume
LVFP: Left ventricular filling pressure
6MWT: 6-Minutes’ walk test
EQ-5D: EuroQol questionnaire
ARBs: Angiotensin receptor blockers
