# Supplementary material for: Soluble guanylate cyclase stimulators for heart failure: a network meta-analysis and subgroup analyses of reduced and preserved ejection fraction
Source: Egypt Heart J. 2024 Jan 24;76:6. doi: 10.1186/s43044-024-00437-x (PMC10808080; doi:10.1186/s43044-024-00437-x)
Supplement: Supplementary file 1 — Additional file 1. Supplementary tables and figures. [file 43044_2024_437_MOESM1_ESM.docx]

**Supplementary material:**

**Contents:**

**Tables.**Table S1: Search strategy.

**Figures.**

Figure S1: Network meta-analysis of cardiovascular mortality/HF hospitalization in HFpEF patients.

Figure S2: Network meta-analysis of all-cause mortality.

Figure S3: Network meta-analysis of any adverse event.

Figure S4: Network meta-analysis of any serious adverse events.

Figure S5: Network meta-analysis of any adverse event leading to drug discontinuation.

Figure S6: Network meta-analysis of syncope.

Figure S7: Network meta-analysis of AKI.

Figure S8: Network meta-analysis of hypotension.

Figure S9: Network meta-analysis of any adverse events in HFrEF patients.

Figure S10: Network meta-analysis of any adverse events in HFpEF patients.

Figure S11: Network meta-analysis of any serious adverse events in HFrEF patients.

Figure S12: Network meta-analysis of any serious adverse events in HFpEF patients.

Figure S13: Network meta-analysis of any adverse event leading to drug discontiuations in HFrEF patients.

Figure S14: Network meta-analysis of any adverse event leading to drug discontiuations in HFpEF patients.

Figure S15: Network meta-analysis syncope in HFrEF patients.

Figure S16: Network meta-analysis syncope in HFpEF patients.

Figure S17: Network meta-analysis AKI in HFrEF patients.

Figure S18: Network meta-analysis AKI in HFpEF patients.

Figure S19: Network meta-analysis of all-cause mortality in HFrEF patients.

Figure S20: Network meta-analysis of hypotension in HFpEF patients.

Figure S20: Network meta-analysis of hypotension in HFrEF patients.

| Database | Search Terms | Search Field | Search Results |
| --- | --- | --- | --- |
| Pubmed | ("heart failure" OR "cardiac failure" OR HFrEF OR HFpEF) AND ("guanylate cyclase stimulator" OR riociguat OR vericiguat OR praliciguat) | All Field | 242 |
| Cochrane | #1 (heart failure):ti,ab,kw OR (cardiac failure):ti,ab,kw OR (hfref):ti,ab,kw OR (hfpef):ti,ab,kw 44198  #2 (guanylate cyclase stimulator):ti,ab,kw OR (riociguat):ti,ab,kw OR (vericiguat):ti,ab,kw OR (praliciguat):ti,ab,kw 343  #3 #1 AND #2 110 | All Field | 110 |
| WOS | ("heart failure" OR "cardiac failure" OR HFrEF OR HFpEF) AND ("guanylate cyclase stimulator" OR riociguat OR vericiguat OR praliciguat) | All Field | 405 |
| SCOPUS | TITLE-ABS-KEY ( ( "heart failure"  OR  "cardiac failure"  OR  hfref  OR  hfpef )  AND  ( "guanylate cyclase stimulator"  OR  riociguat  OR  vericiguat  OR  praliciguat ) ) | Title, Abstract, Keywords | 640 |
| EMBASE | #3.  #1 AND #2  #2.  'guanylate cyclase stimulator':ti,ab,kw OR       riociguat:ti,ab,kw OR vericiguat:ti,ab,kw OR       praliciguat:ti,ab,kw  #1.  'heart failure':ti,ab,kw OR 'cardiac       failure':ti,ab,kw OR hfref:ti,ab,kw OR       hfpef:ti,ab,kw | All Field | 367 |

Table S1: Search Strategy.


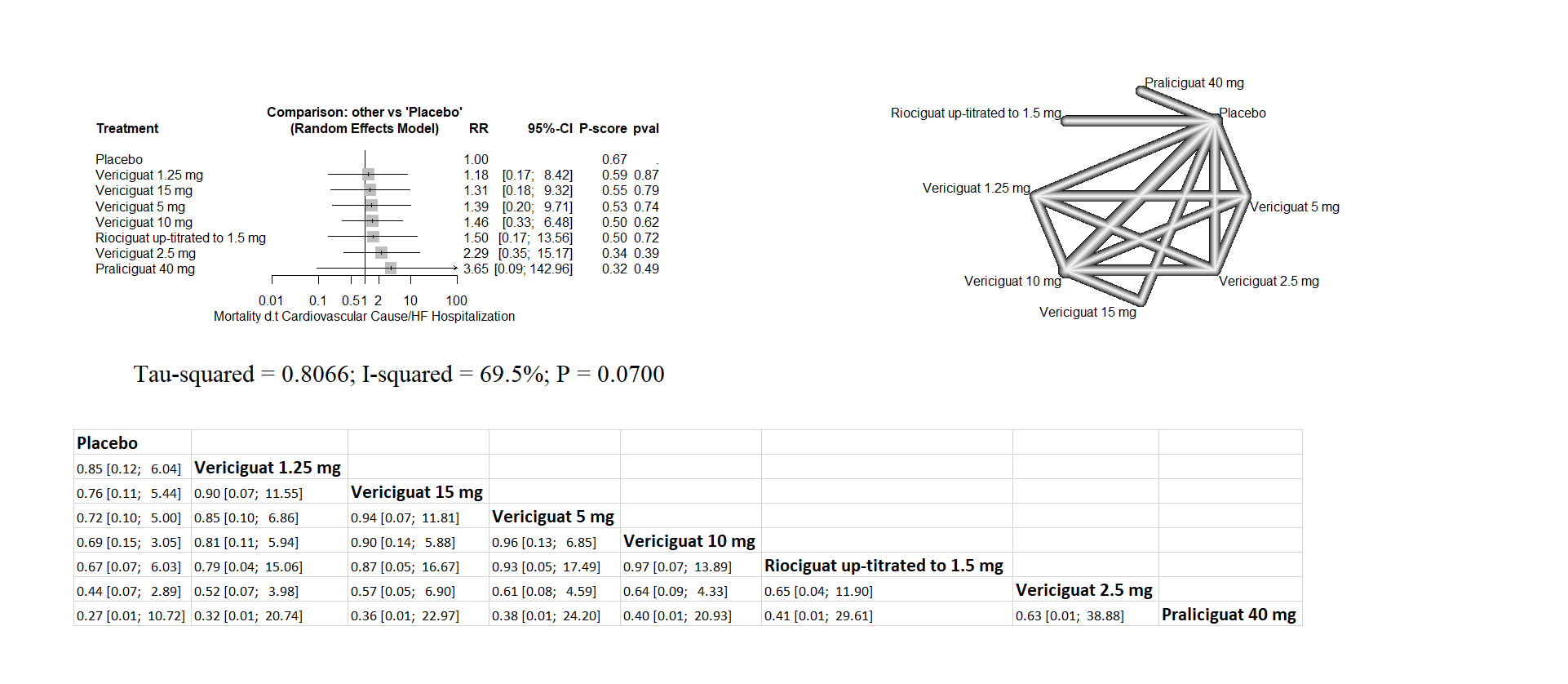


Figure S1: Network meta-analysis of cardiovascular mortality/HF hospitalization in HFpEF patients.


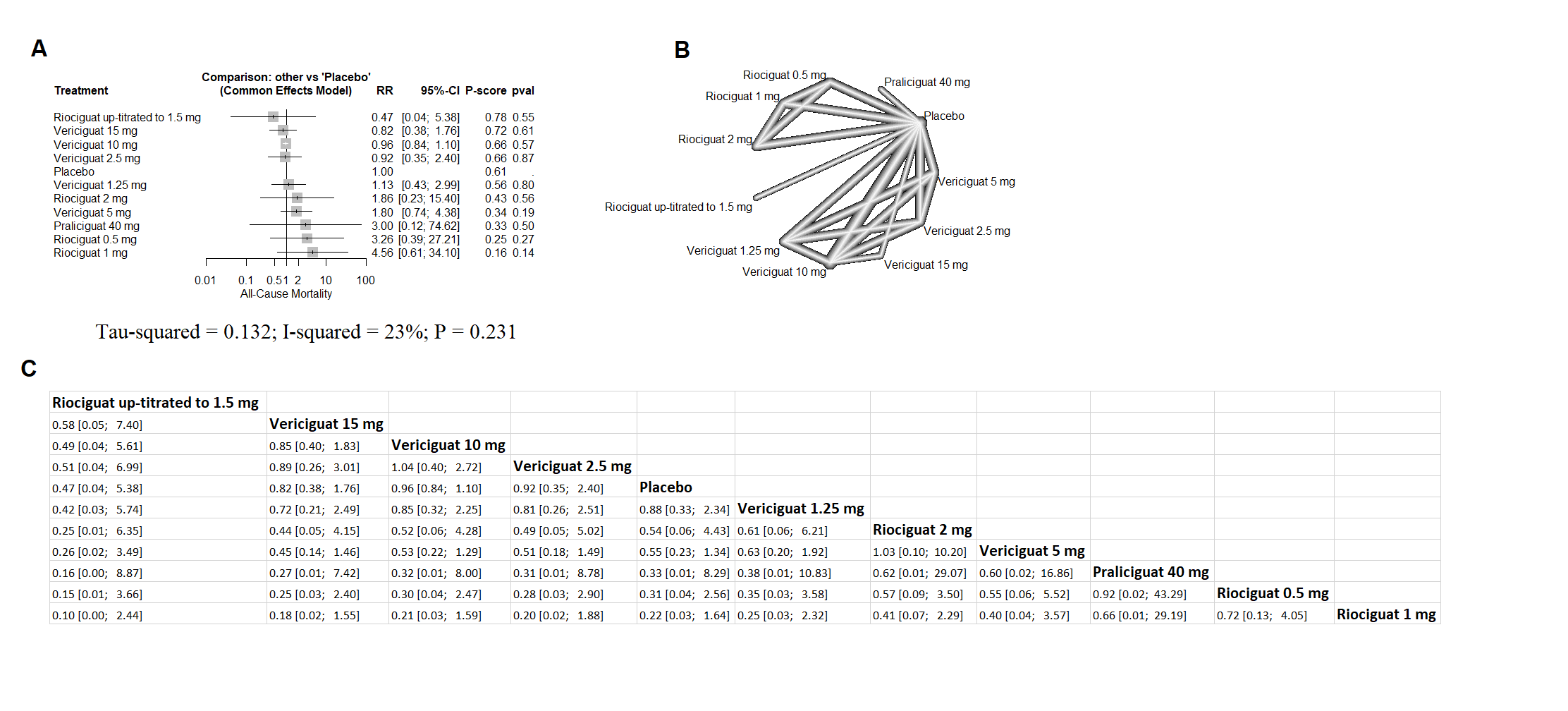


Figure S2: Network meta-analysis of all-cause mortality.


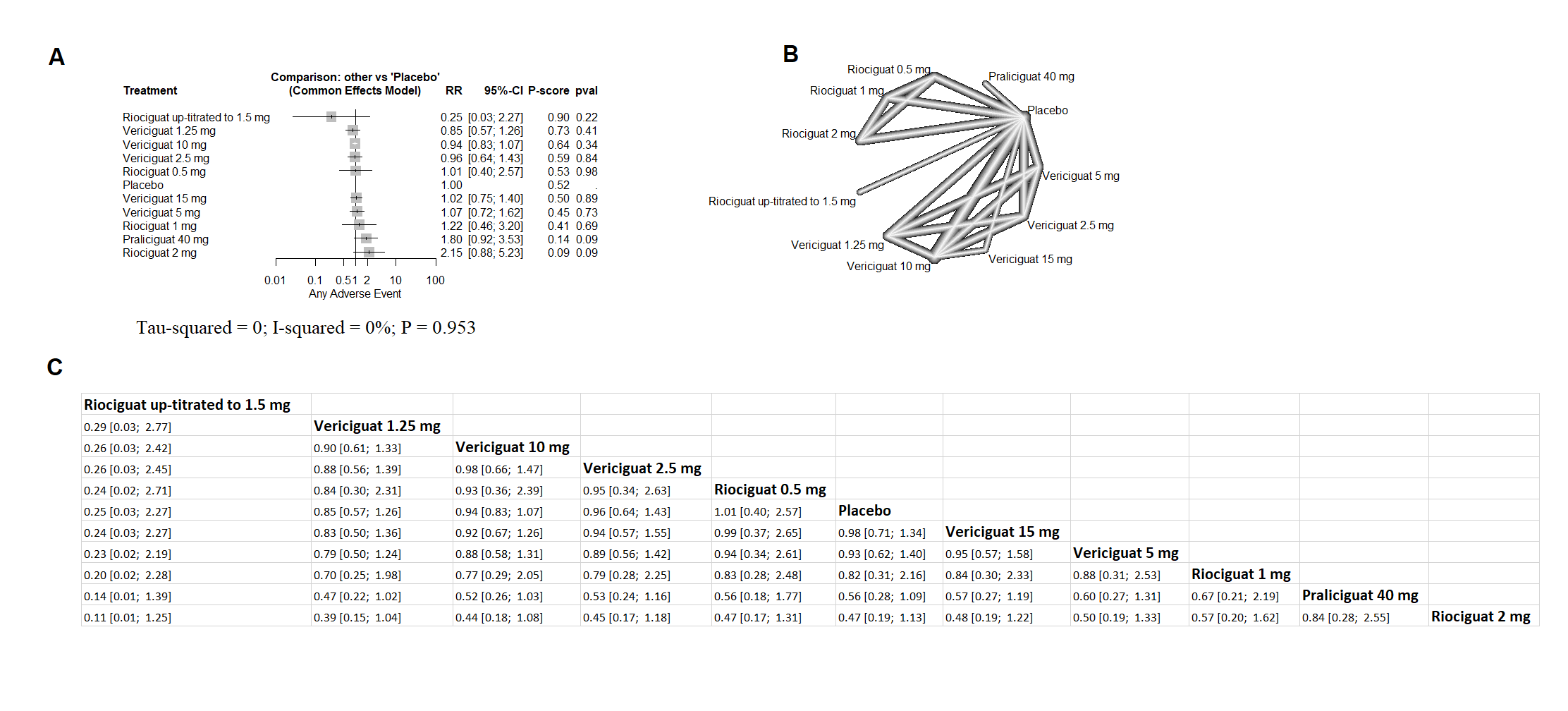


Figure S3: Network meta-analysis of any adverse event.


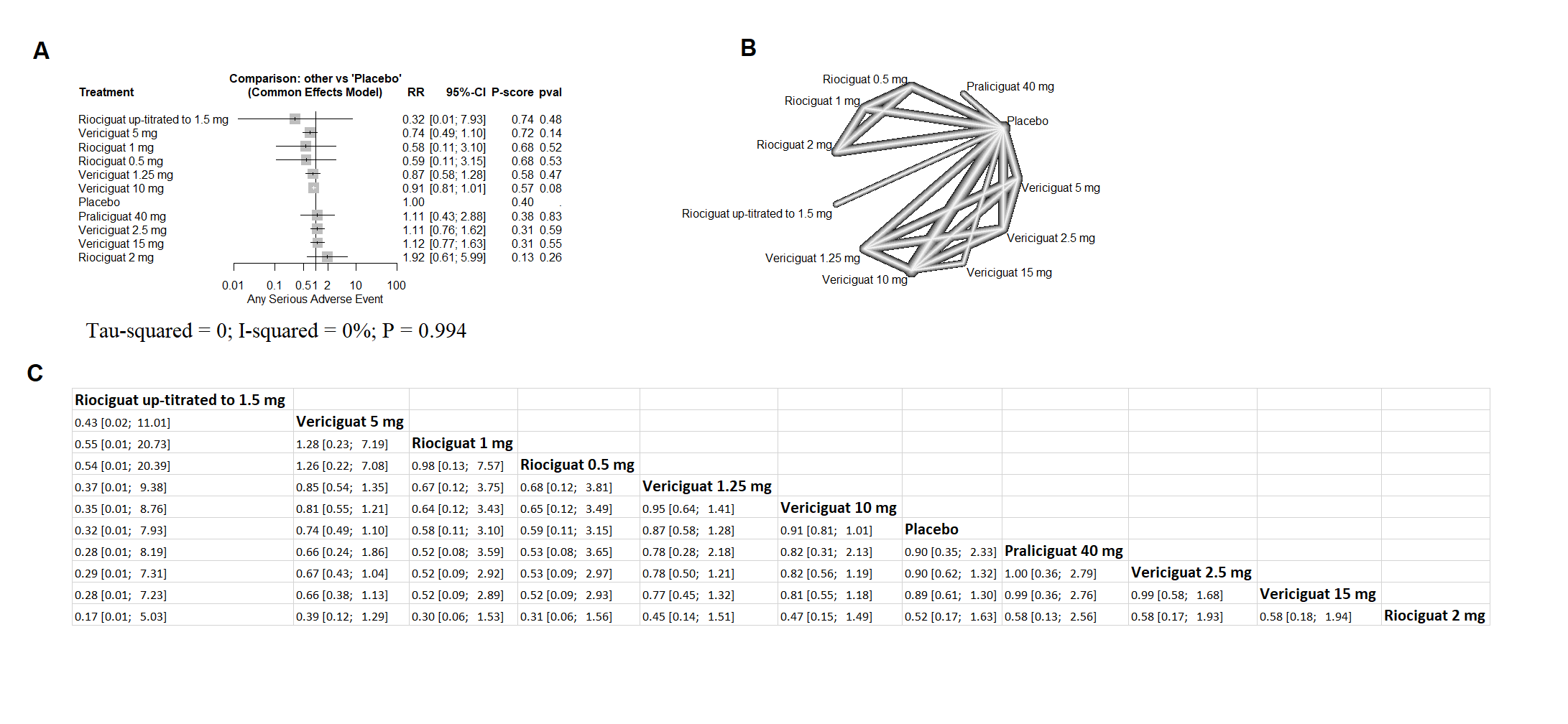


Figure S4: Network meta-analysis of any serious adverse events.


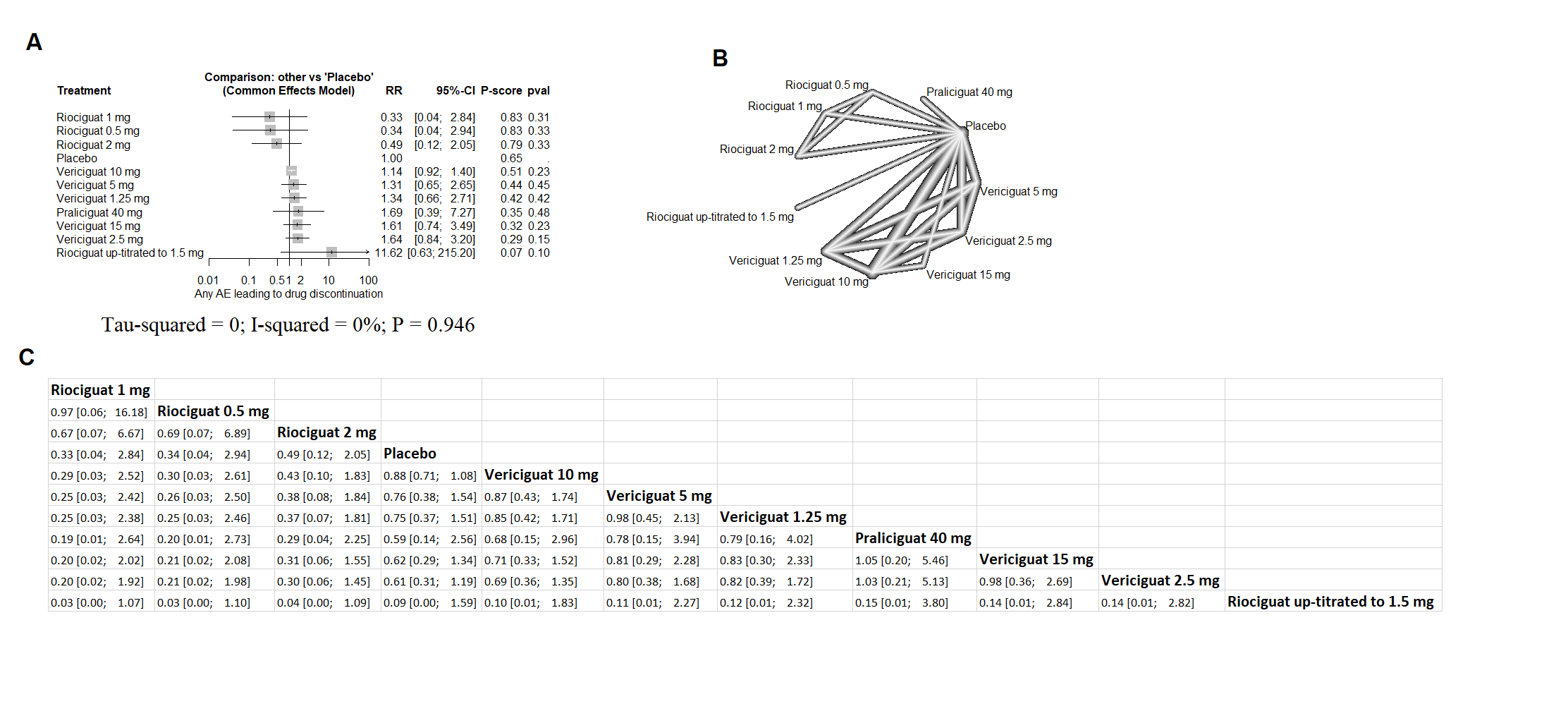


Figure S5: Network meta-analysis of any adverse event leading to drug discontinuation.


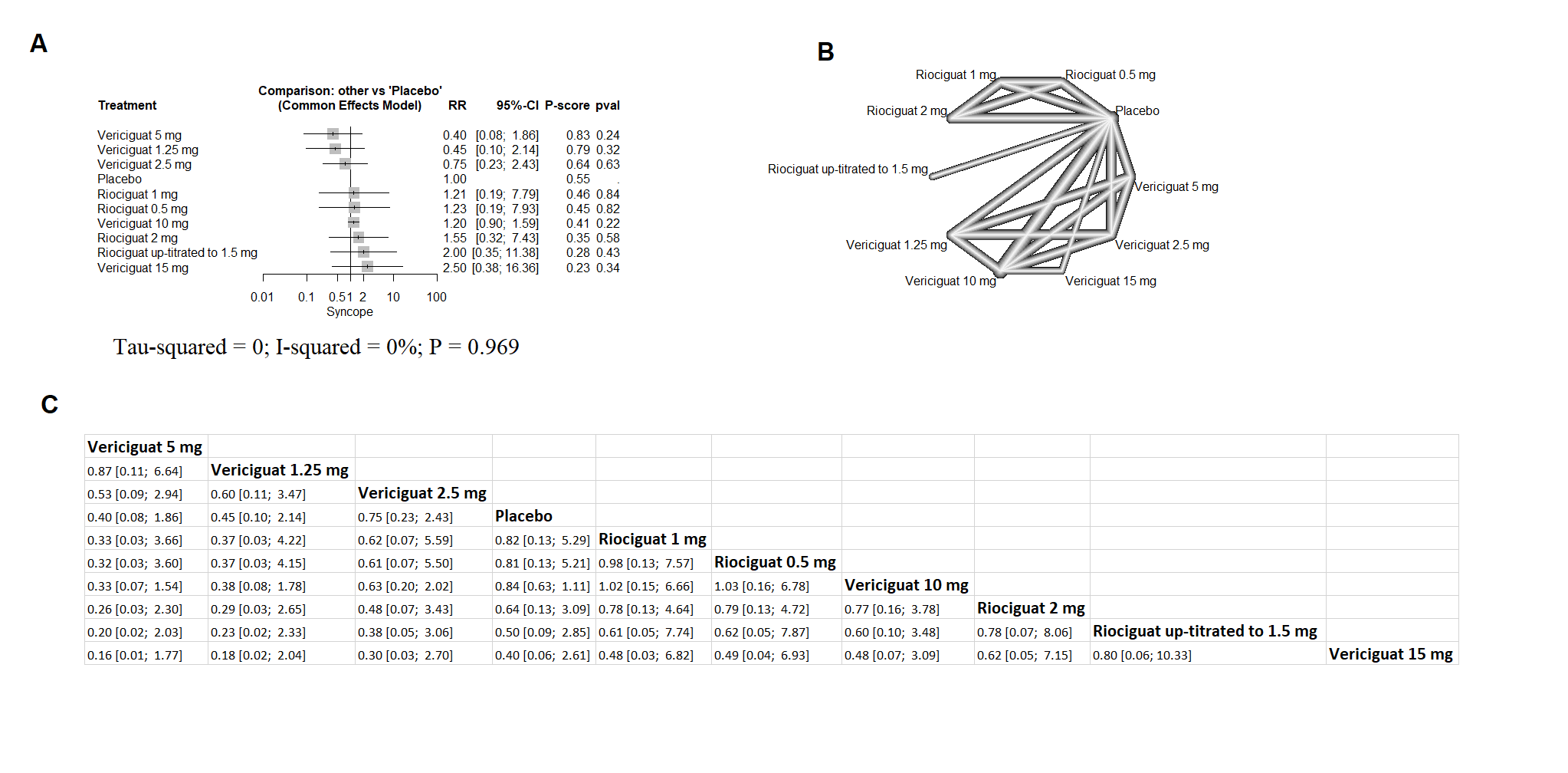


Figure S6: Network meta-analysis of syncope.


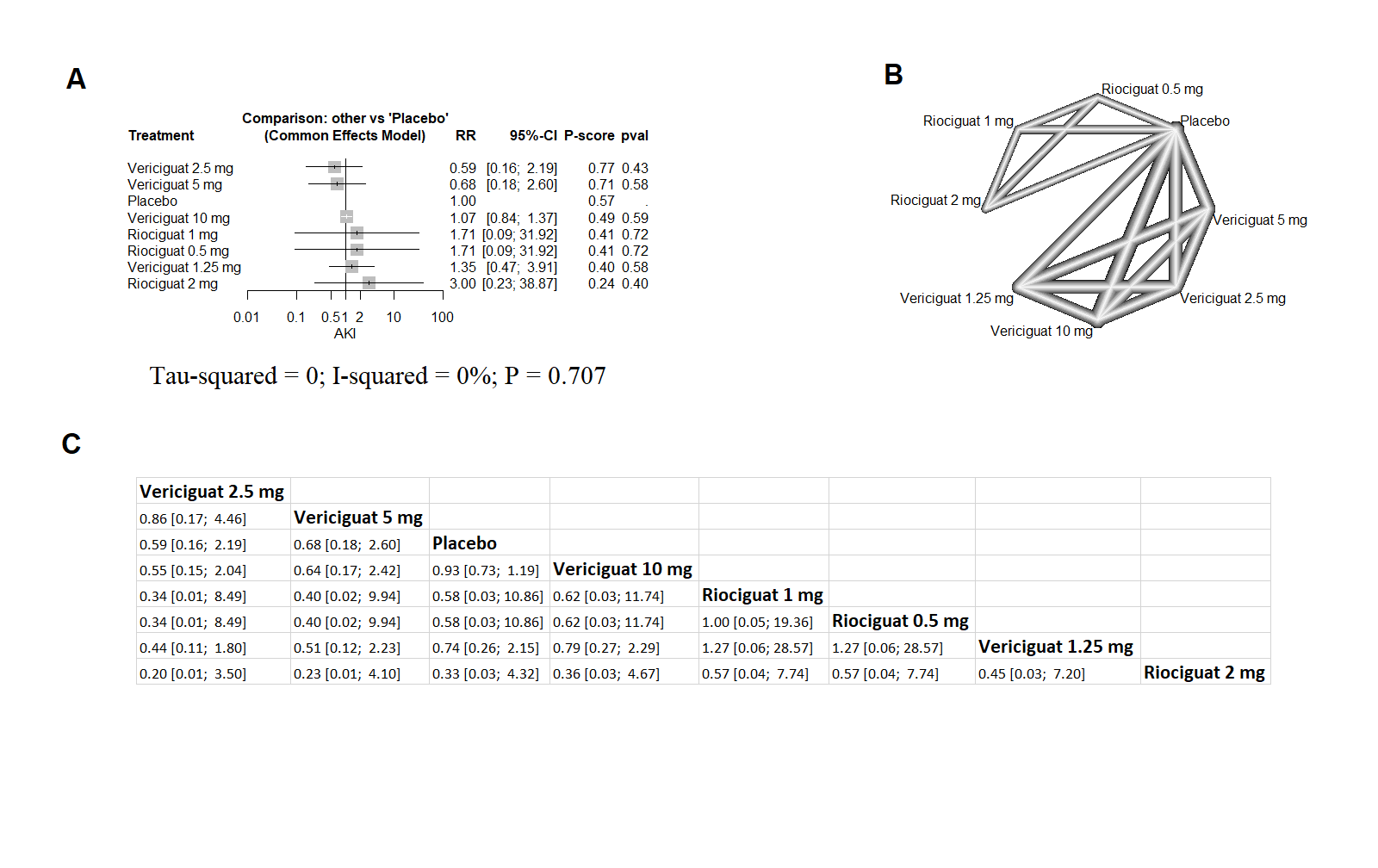


Figure S7: Network meta-analysis of AKI.


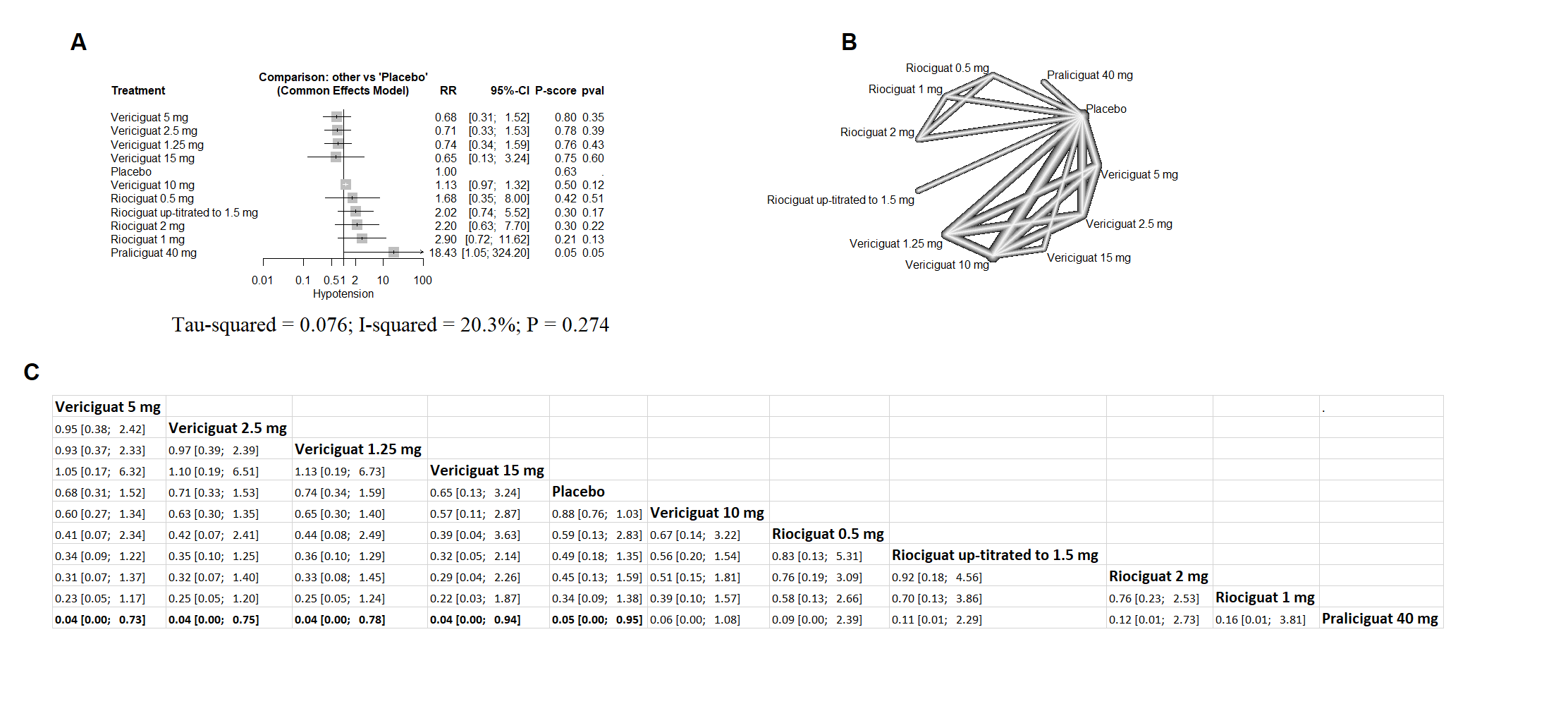


Figure S8: Network meta-analysis of hypotension.


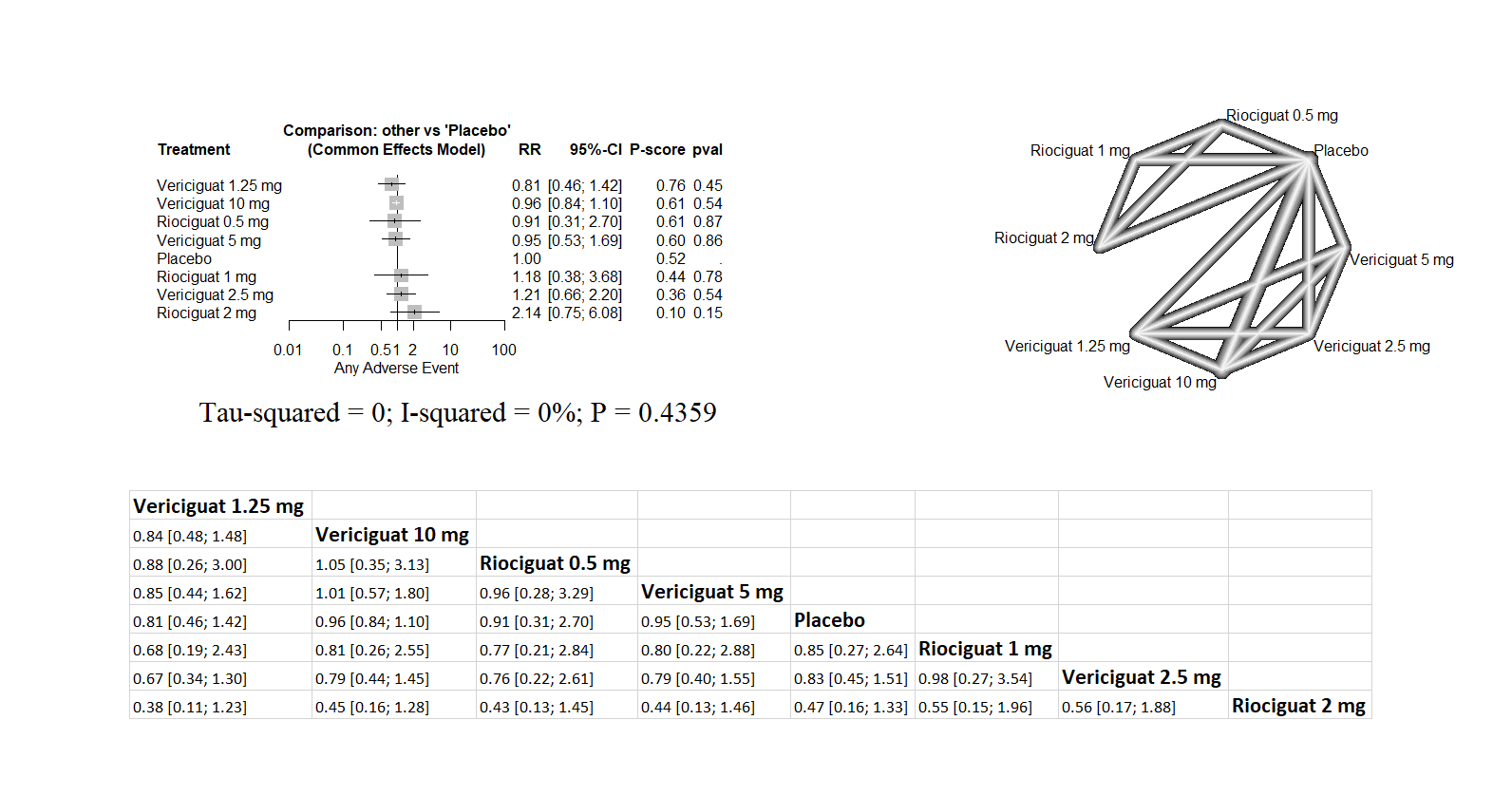


Figure S9: Network meta-analysis of any adverse events in HFrEF patients.


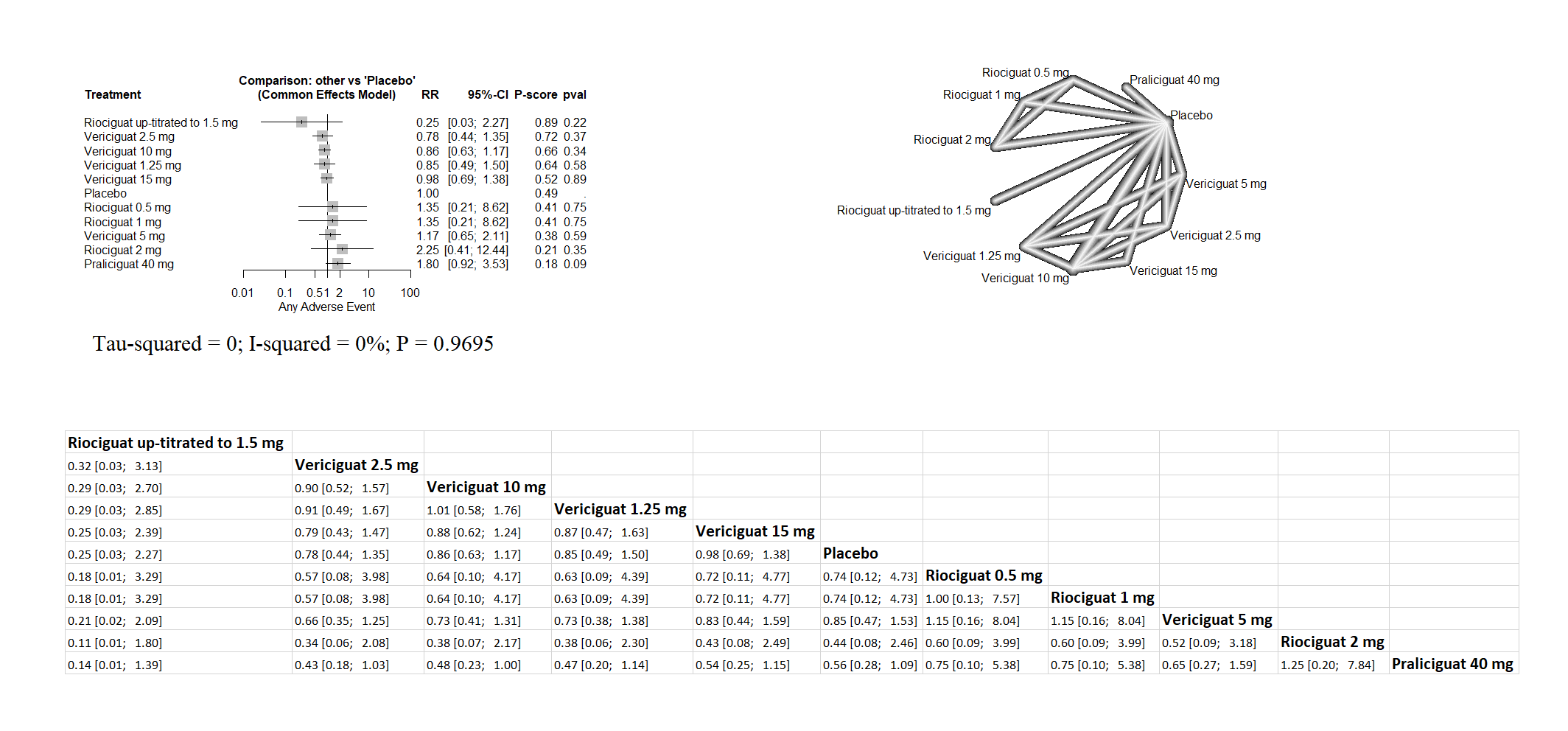


Figure S10: Network meta-analysis of any adverse events in HFpEF patients.


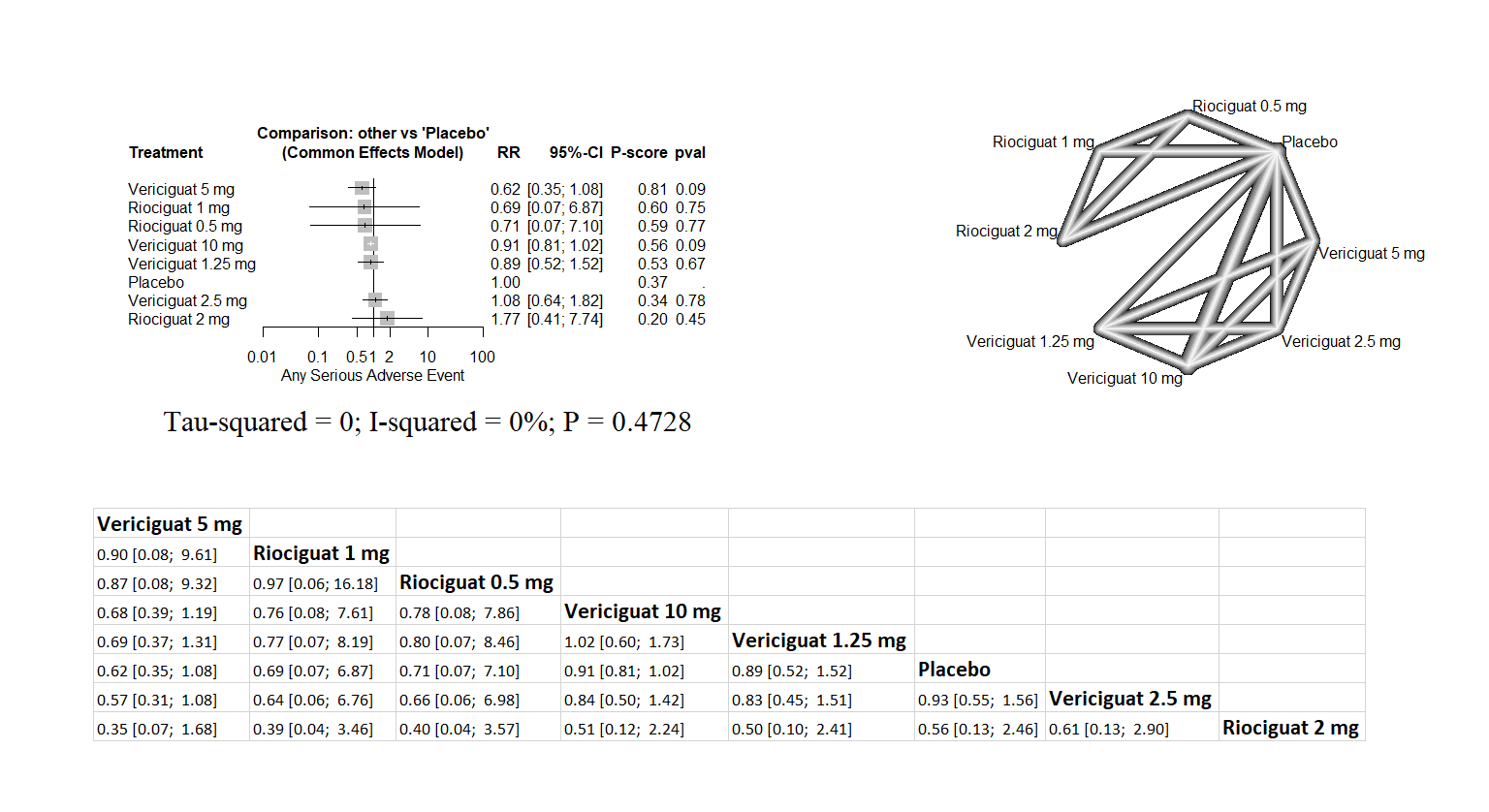


Figure S11: Network meta-analysis of any serious adverse events in HFrEF patients.


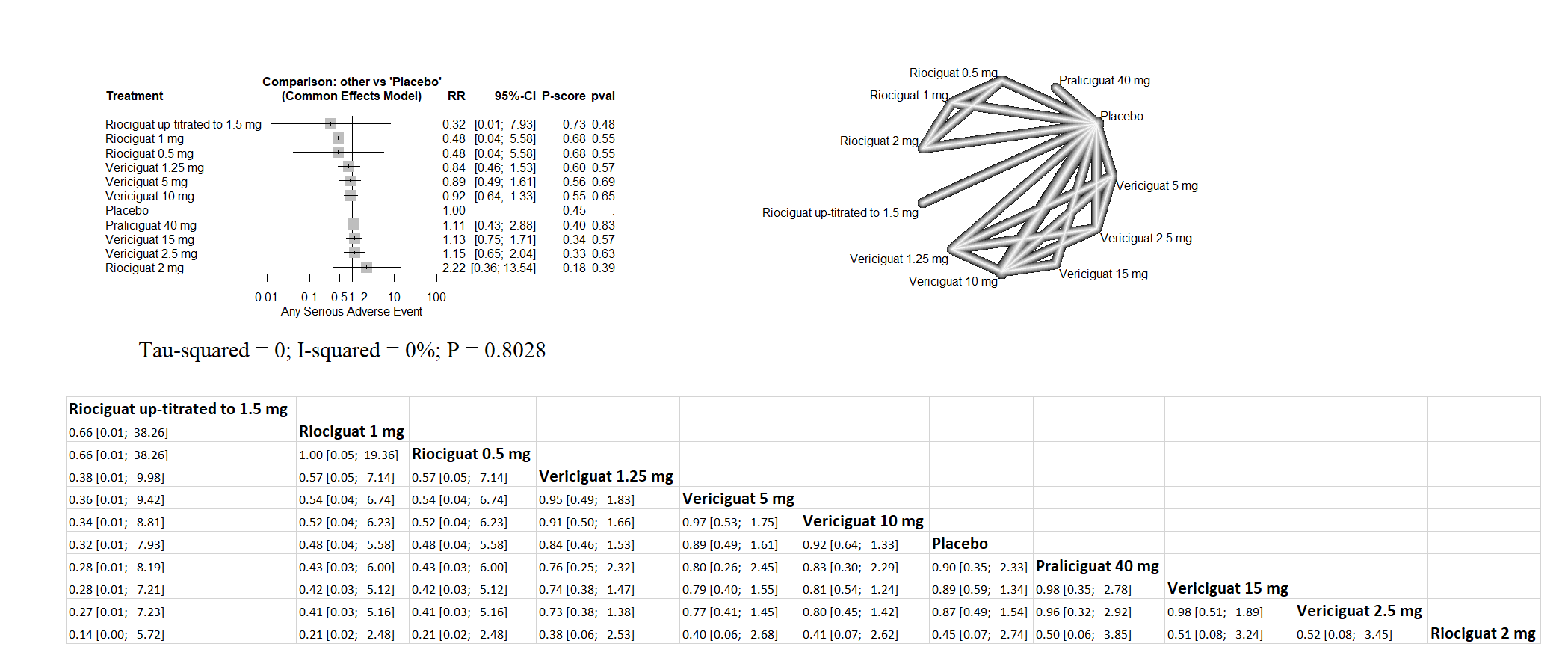


Figure S12: Network meta-analysis of any serious adverse events in HFpEF patients.


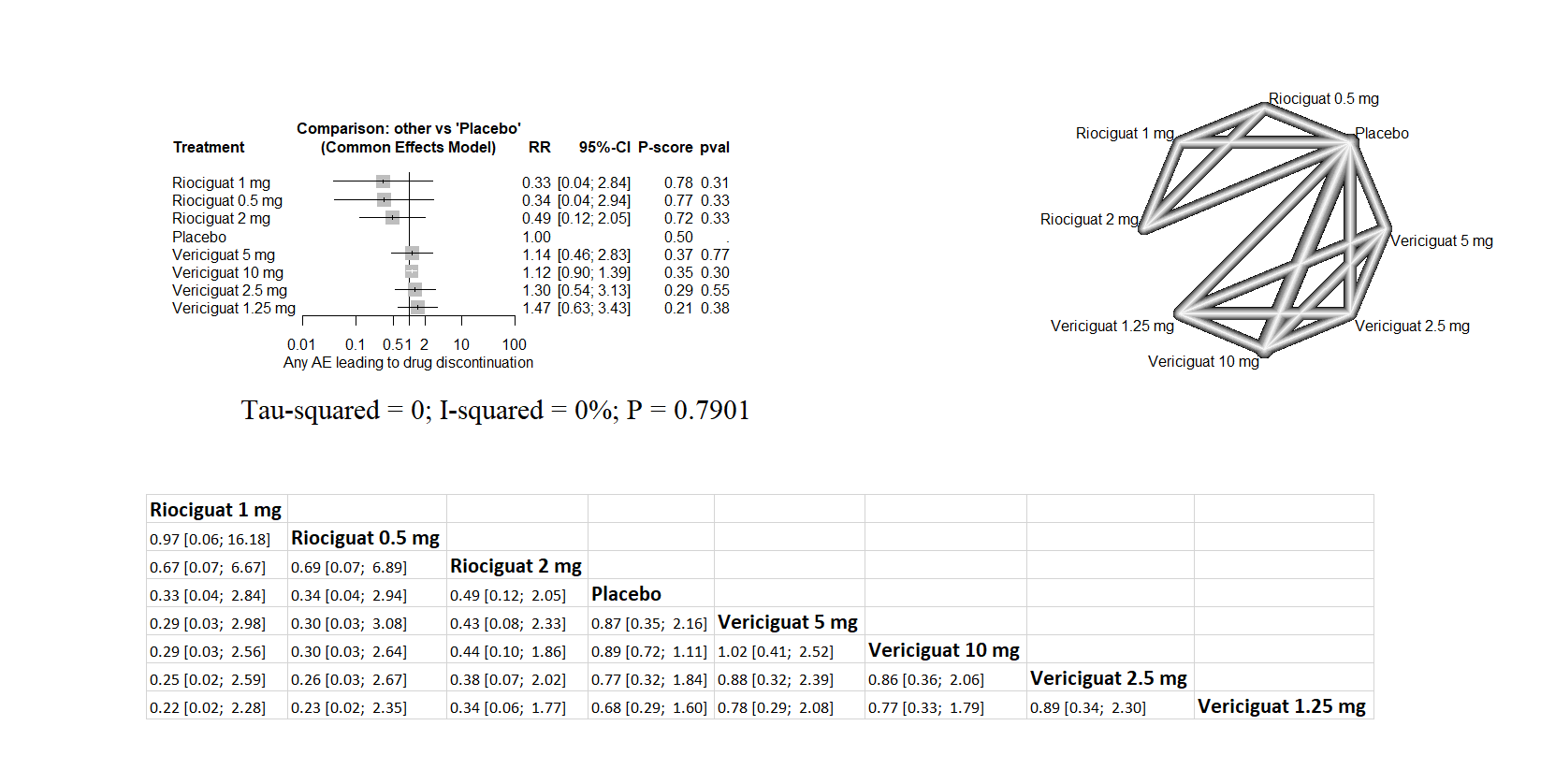


Figure S13: Network meta-analysis of any adverse event leading to drug discontiuations in HFrEF patients.


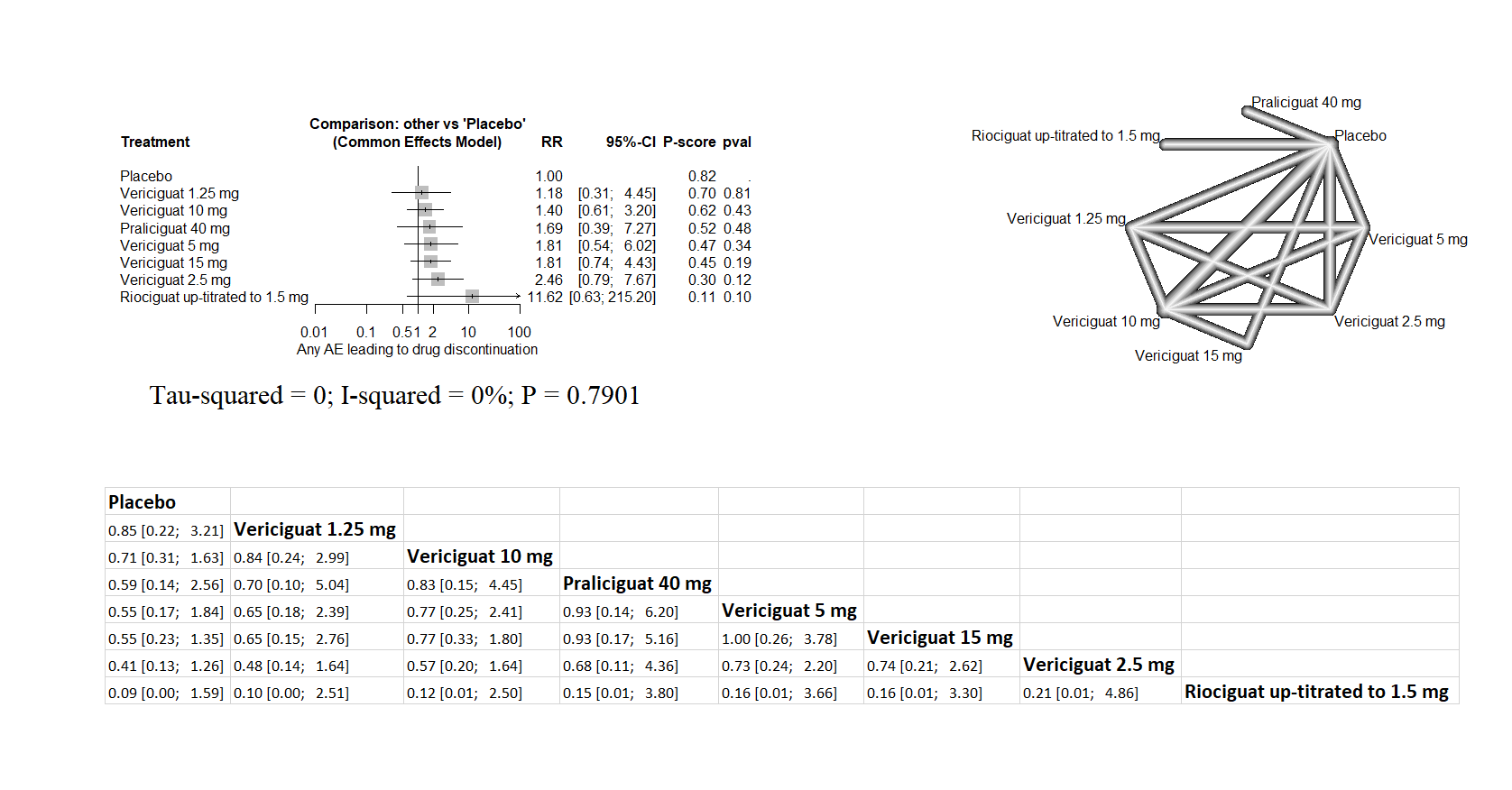


Figure S14: Network meta-analysis of any adverse event leading to drug discontiuations in HFpEF patients.


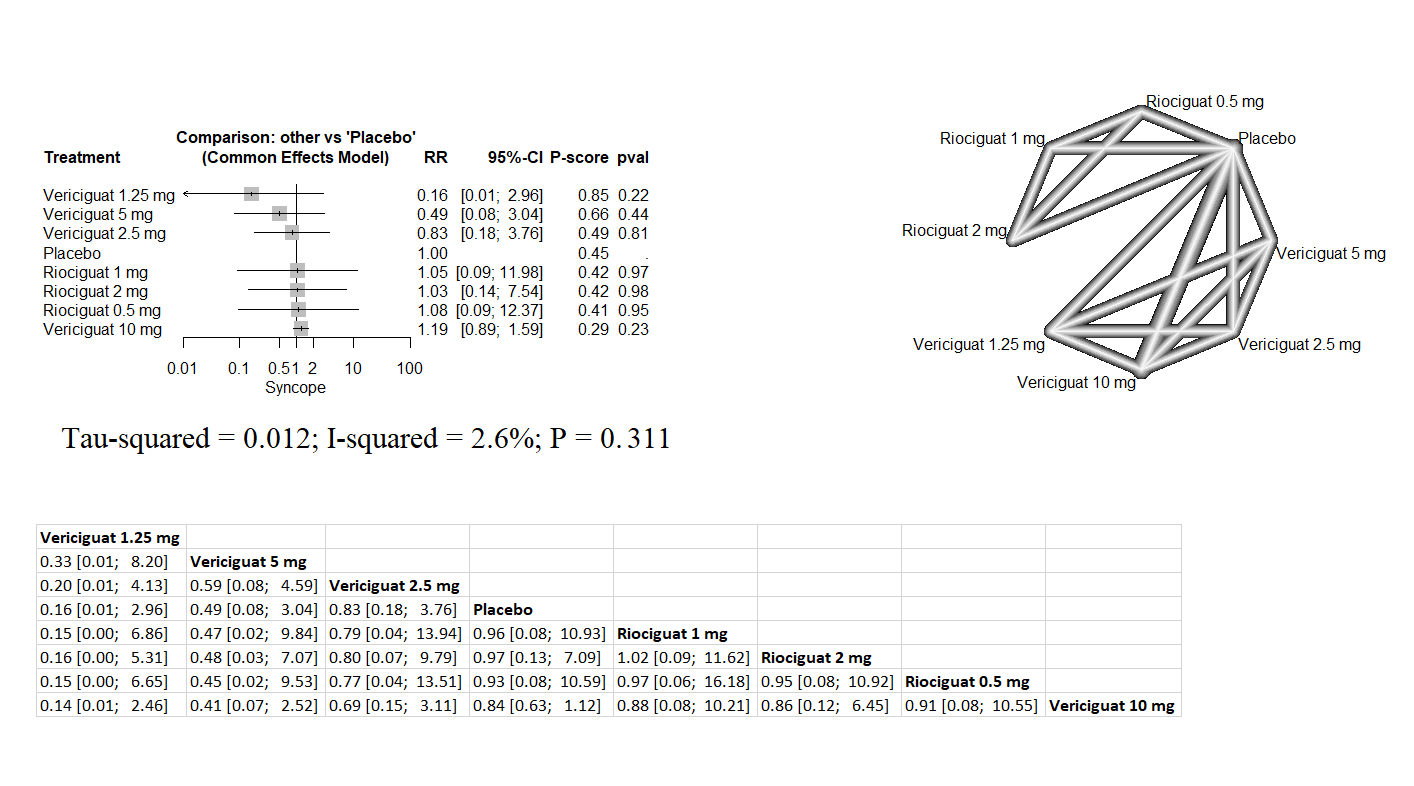


Figure S15: Network meta-analysis syncope in HFrEF patients.


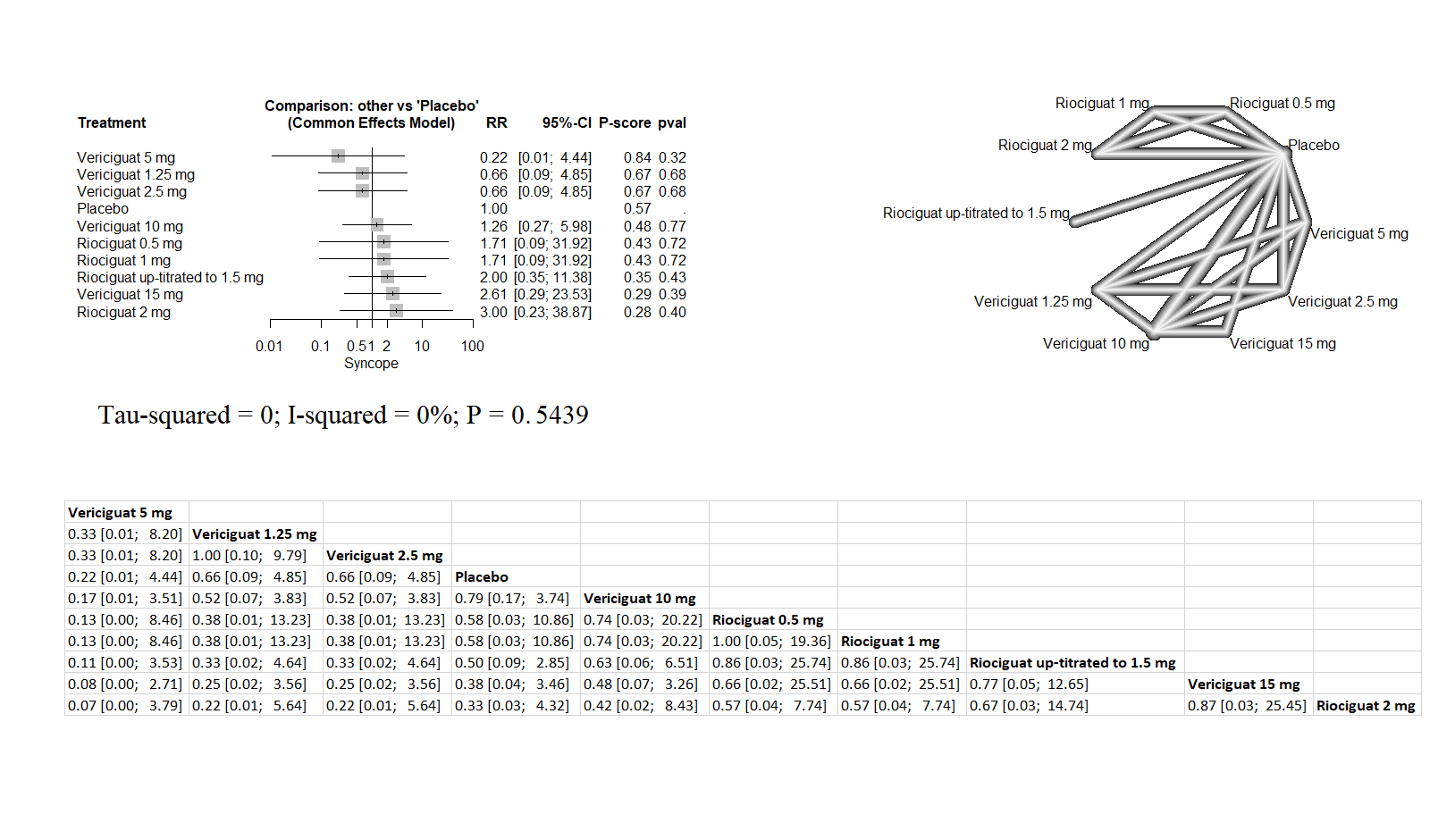


Figure S16: Network meta-analysis syncope in HFpEF patients.


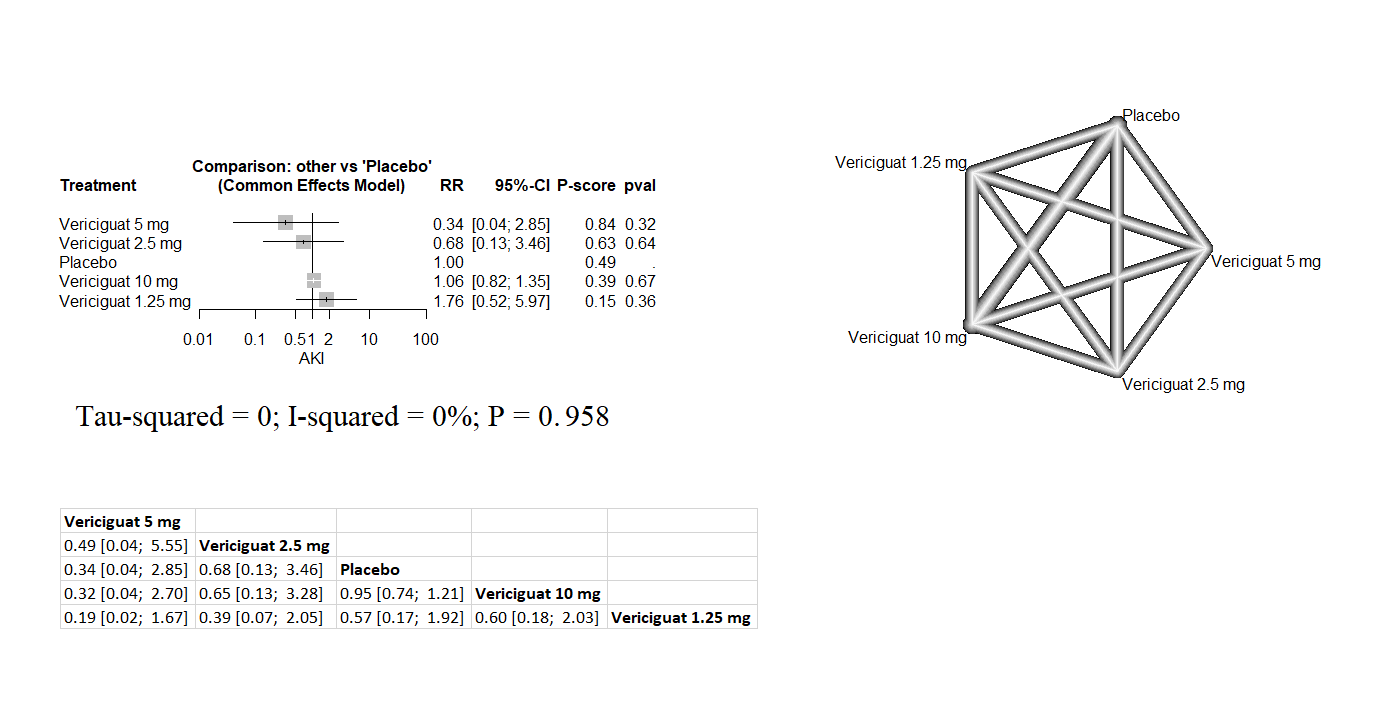


Figure S17: Network meta-analysis AKI in HFrEF patients.


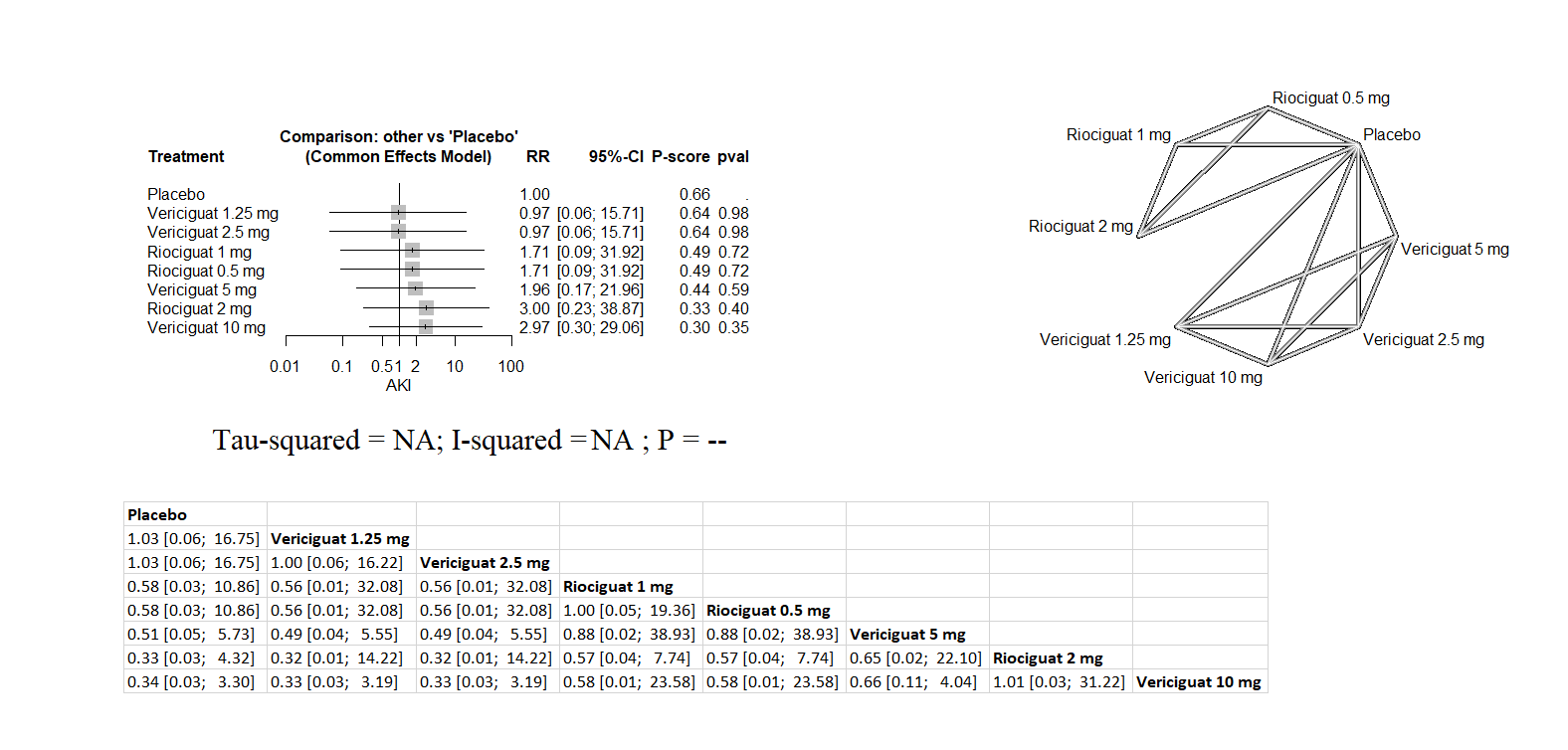


Figure S18: Network meta-analysis AKI in HFpEF patients.


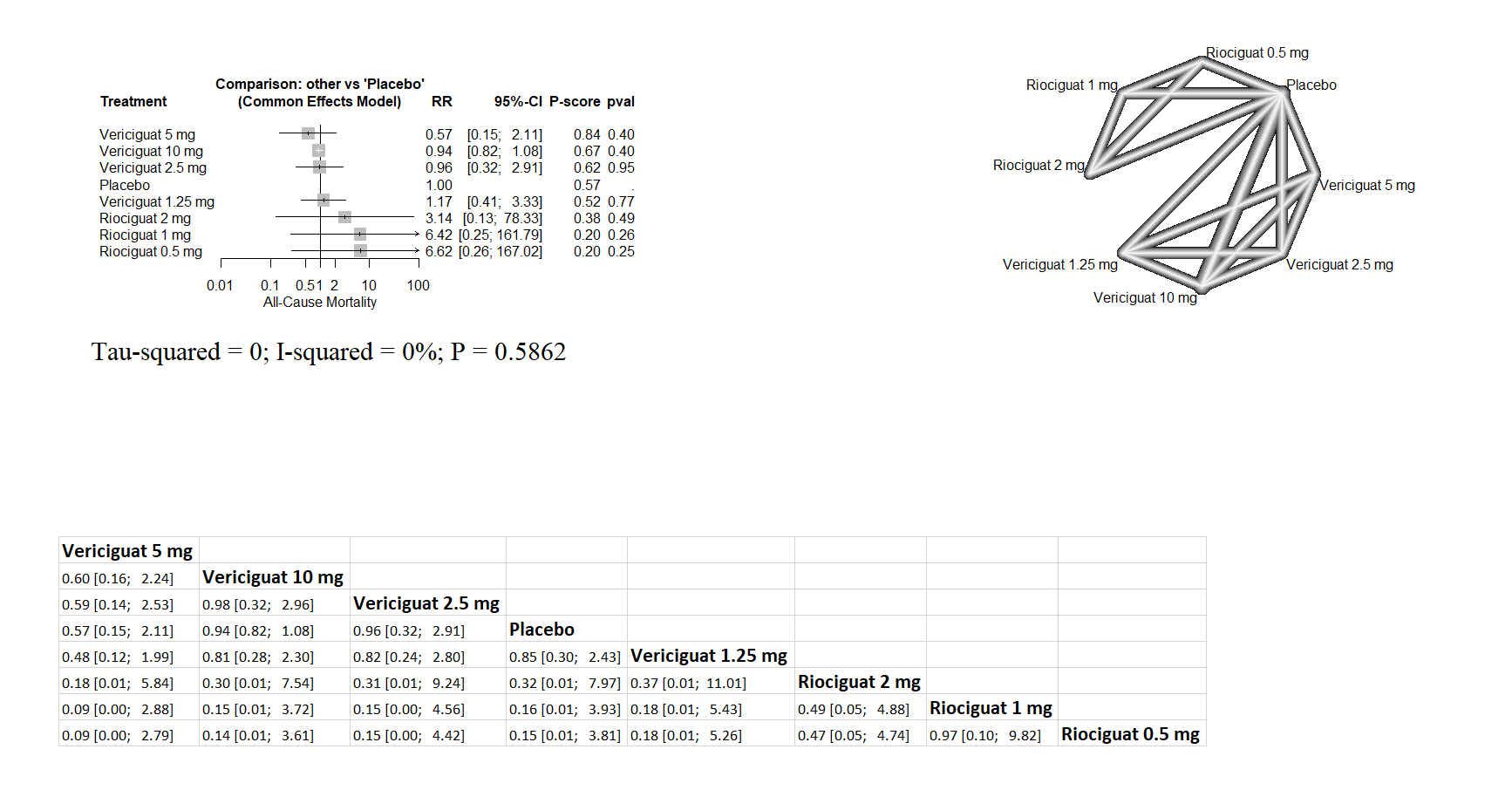


Figure S19: Network meta-analysis of all-cause mortality in HFrEF patients.


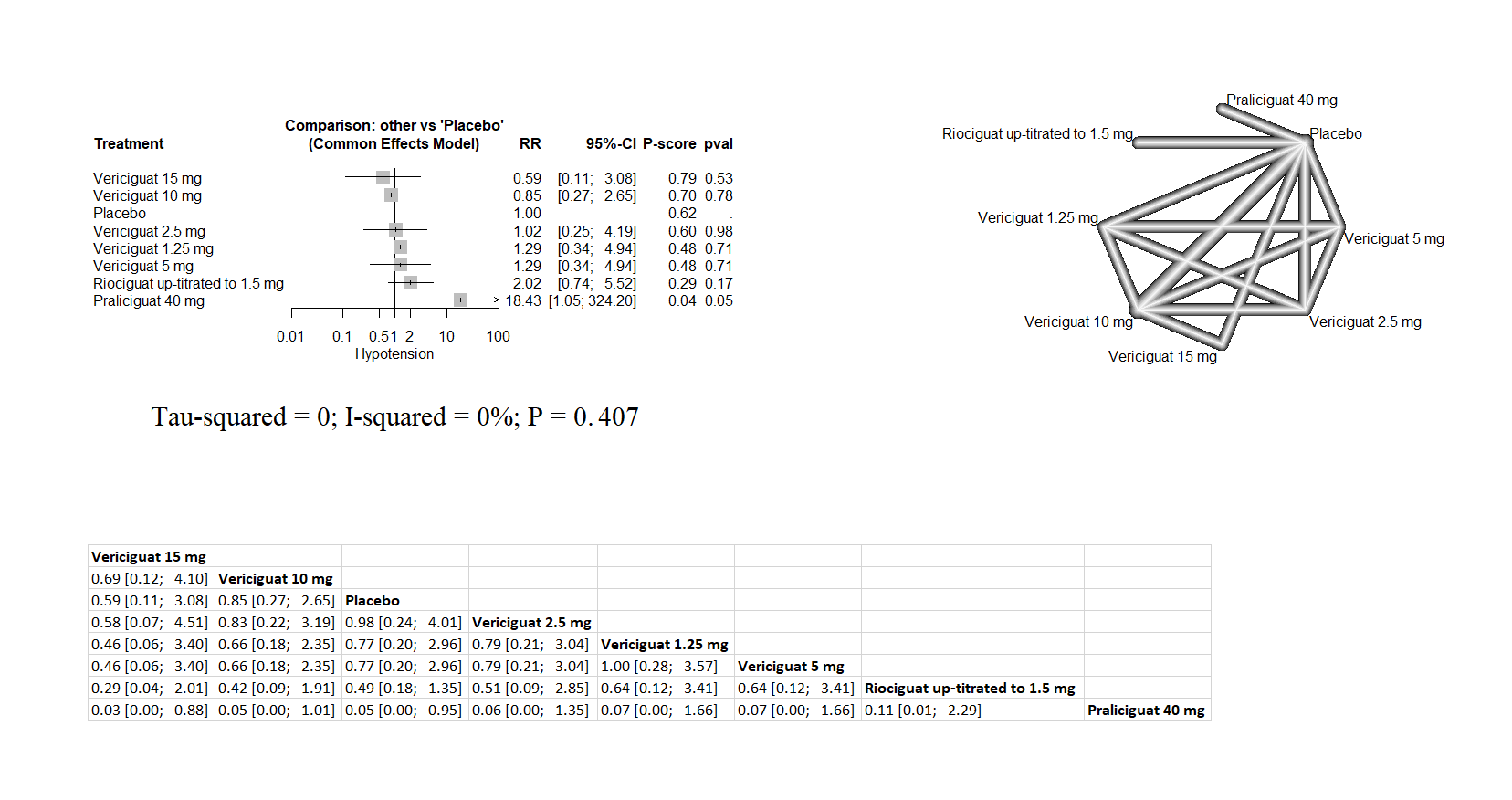


Figure S20: Network meta-analysis of hypotension in HFpEF patients.


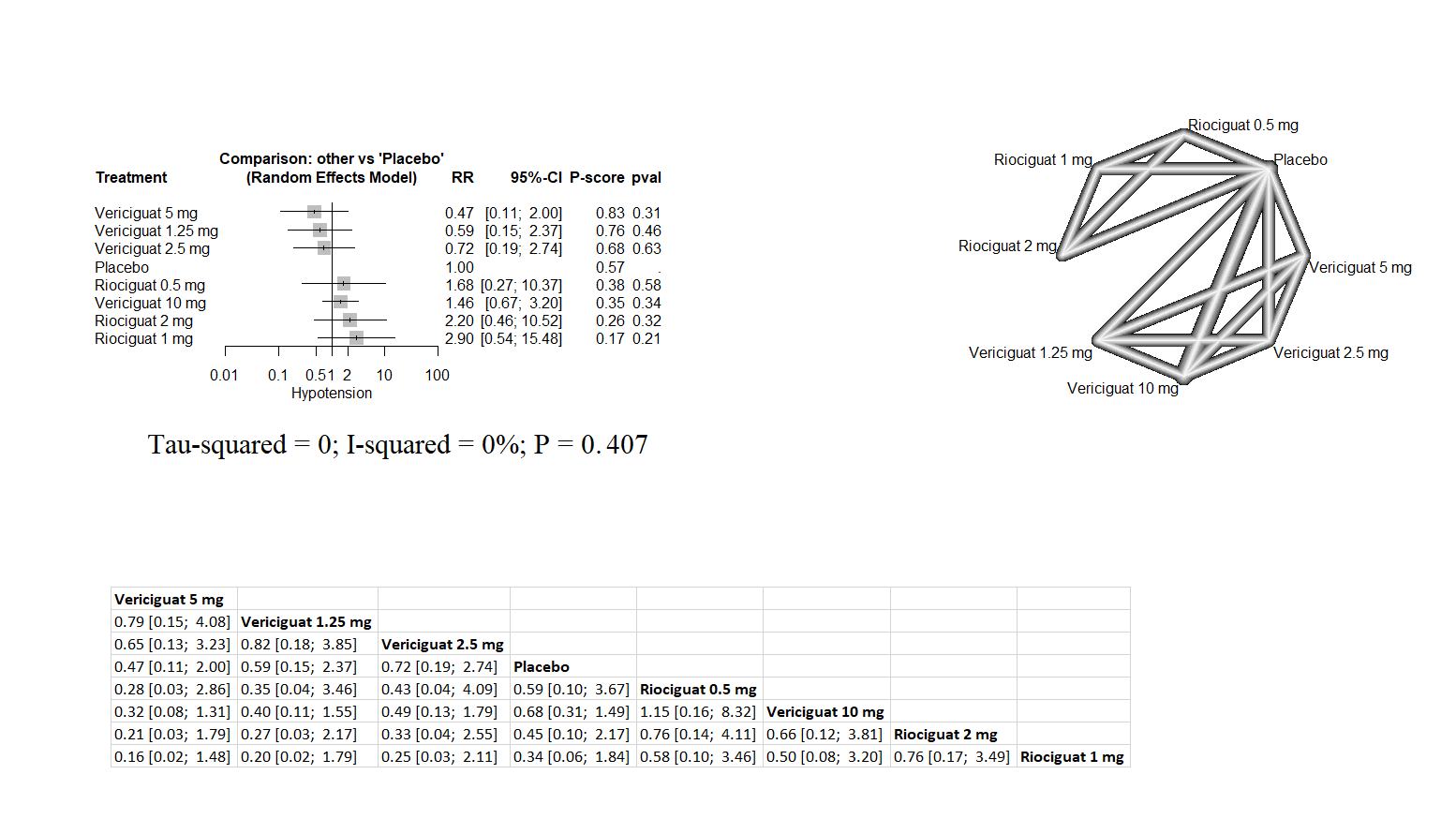


Figure S21: Network meta-analysis of hypotension in HFrEF patients.
